# The TORC2-Dependent Signaling Network in the Yeast *Saccharomyces cerevisiae*

**DOI:** 10.3390/biom7030066

**Published:** 2017-09-05

**Authors:** Françoise M. Roelants, Kristin L. Leskoske, Maria Nieves Martinez Marshall, Melissa N. Locke, Jeremy Thorner

**Affiliations:** Division of Biochemistry, Biophysics and Structural Biology and Division of Cell and Developmental Biology, Department of Molecular and Cell Biology, University of California, Berkeley, CA 94720-3202, USA; roelants@berkeley.edu (F.M.R.); kleskoske@berkeley.edu (K.L.L.); nmartinezm@berkeley.edu (M.N.M.M.); mnlocke@berkeley.edu (M.N.L.)

**Keywords:** protein kinase, protein phosphorylation, regulation, metabolic control, mutants

## Abstract

To grow, eukaryotic cells must expand by inserting glycerolipids, sphingolipids, sterols, and proteins into their plasma membrane, and maintain the proper levels and bilayer distribution. A fungal cell must coordinate growth with enlargement of its cell wall. In *Saccharomyces cerevisiae*, a plasma membrane-localized protein kinase complex, Target of Rapamicin (TOR) complex-2 (TORC2) (mammalian ortholog is mTORC2), serves as a sensor and master regulator of these plasma membrane- and cell wall-associated events by directly phosphorylating and thereby stimulating the activity of two types of effector protein kinases: Ypk1 (mammalian ortholog is SGK1), along with a paralog (Ypk2); and, Pkc1 (mammalian ortholog is PKN2/PRK2). Ypk1 is a central regulator of pathways and processes required for plasma membrane lipid and protein homeostasis, and requires phosphorylation on its T-loop by eisosome-associated protein kinase Pkh1 (mammalian ortholog is PDK1) and a paralog (Pkh2). For cell survival under various stresses, Ypk1 function requires TORC2-mediated phosphorylation at multiple sites near its C terminus. Pkc1 controls diverse processes, especially cell wall synthesis and integrity. Pkc1 is also regulated by Pkh1- and TORC2-dependent phosphorylation, but, in addition, by interaction with Rho1-GTP and lipids phosphatidylserine (PtdSer) and diacylglycerol (DAG). We also describe here what is currently known about the downstream substrates modulated by Ypk1-mediated and Pkc1-mediated phosphorylation.

## 1. Introduction

Growth and division of a eukaryotic cell are complex processes coupled to various stimuli, the availability of nutrients, and additional external and internal cues, all of which are tightly regulated at many levels, especially when challenged by suboptimal conditions and other stresses. When it was found that the antibiotic rapamycin was a potent inhibitor of the proliferation of virtually every eukaryotic cell type examined (e.g., fungi, T cells, and tumor cells) [1,2,3], it became clear that the molecular target of rapamycin must be highly conserved and its function critical for cell survival. Indeed, ever since the authentic target of rapamycin (TOR) was first discovered using an elegant genetic approach in budding yeast (*Saccharomyces cerevisiae*) [4,5], TOR has emerged as a universal, centrally important sensor, integrator, and controller of eukaryotic cell growth [6,7]. 

As first demonstrated in yeast [8,9,10,11], TOR is found in all eukaryotic cells in two distinct macromolecular complexes, TOR complex 1 (TORC1) and TOR complex 2 (TORC2). TORC1 is sensitive to inhibition by rapamycin, whereas TORC2 is normally insensitive [12,13,14,15]. The catalytic subunit in both TORC1 and TORC2 is the very large TOR polypeptide; metazoans possess a single TOR-encoding gene (human mTOR, 2549 residues), whereas budding yeast [4], fission yeast [16], and other fungi [17] encode two TOR proteins, Tor1 and Tor2 (2470 and 2474 residues, respectively, in *S. cerevisiae*).

## 2. TORC2 Structure and Function

Only Tor2 can serve as the catalytic subunit in yeast TORC2, whereas TORC1 is functional when its catalytic subunit is either Tor1 or Tor2 [10,11]. The small β-propeller (“WD40 repeat”) protein Lst8 is present in both TORC1 and TORC2, presumably because, as shown for its mammalian ortholog (mLST8), it binds tightly to and greatly stabilizes the TOR kinase fold [18,19,20]. Aside from Lst8, however, the other known subunits in yeast TORC2, namely Avo1 (mammalian ortholog is mSIN1), Avo2 (appears to be fungal-specific), Avo3/Tsc11 (mammalian ortholog is Rictor), Bit2 and Bit61 (mammalian counterparts are Protor1/PRR5 and Protor2/PRR5L), and Slm1 and Slm2 (also fungal-specific), are all separate and distinct from those in TORC1, as reviewed in detail elsewhere [5,17,21]. Structural, genetic, and biochemical analyses have revealed that TORC2 is only insensitive to rapamycin because the C-terminus of Avo3 blocks the ability of rapamycin-bound FKBP12 (Fpr1 in *S. cerevisiae*) to bind to the FKBP12-rapamycin binding (FRB) domain of Tor2; deleting a portion of the Avo3 C-terminus renders TORC2 sensitive to rapamycin inhibition [15]. In a yeast cell where such an *avo3* truncation (*avo3∆C*) is combined with a dominant point mutation (*TOR1-1*) in the FRB domain of Tor1 that blocks its association with rapamycin-Fpr1 [4], TORC2 can be uniquely inhibited by addition of rapamycin [15].

Using budding yeast (*S. cerevisiae*) as the experimental organism, it has been shown by us [22,23,24,25,26] and others [27,28,29,30,31] that TORC2 plays an essential role in sensing the status of the plasma membrane (PM) and controlling the reactions that ensure PM homeostasis. In addition to influencing reactions that affect PM lipid and protein composition, TORC2 action also modulates assembly and function of the actin cytoskeleton and actin-driven endocytosis [26,32,33,34].

## 3. TORC2 Effectors 

**Figure 1:**
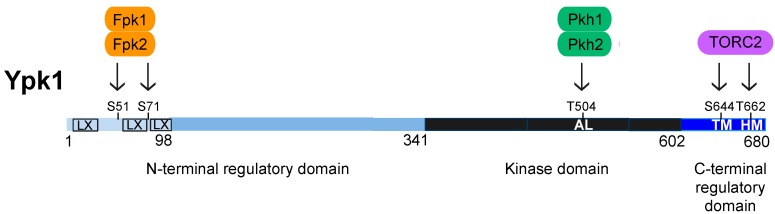
Schematic depiction of the primary structure of Ypk1. Catalytic domain (black) and N- (light blue) and C-terminal (dark blue) regulatory elements are indicated. Shading reflects percent sequence identity between Ypk1 (680 residues) and the corresponding segment in its paralog Ypk2 (677 residues): 1–98, 22% (faint blue); 99–341, 62% (medium blue); 342–602, 90% (black); and, 603–680, 73% (dark blue). Abbreviations: LX, low-complexity sequences predicted by UnitProt [50] and/or SMART [51] databases; AL, activation loop Thr (T504), phosphorylated by Pkh1 and, less efficiently, by Pkh2 [46]; TM, turn motif Ser (S644), phosphorylated by TORC2; and, HM, hydrophobic motif Thr (T662), phosphorylated by TORC2. Two N-terminal residues phosphorylated by Fpk1 and, less efficiently, by Fpk2 [52] are also shown.

**Figure 2:**
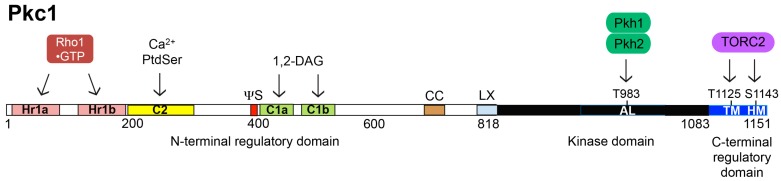
Schematic depiction of the primary structure of Pkc1. Catalytic domain (black) and N- (white) and C-terminal (dark blue) regulatory elements are indicated. Abbreviations: Hr1 (pink), Homology region 1, a compact helical bundle, also referred to as an ACC (anti-parallel coiled-coil) finger domain, which binds small GTPases of the Rho family; C2 (yellow), Conserved region 2, an 8-stranded β-sandwich rich in acidic residues, also referred to as a CalB (Ca^2+^-binding) domain, which binds to phospholipids, especially PtdSer, in a Ca^2+^-dependent manner; ΨS (red), pseudosubstrate sequence, a short sequence that lacks a phosphoacceptor residue, but otherwise possesses the features of the optimal Pkc1 consensus phosphoacceptor site, which binds to and occludes the catalytic pocket in the kinase domain, thereby keeping the enzyme inactive; C1 (green), Conserved region 1, a Cys-rich Zn^2+^-binding fold with the capacity to bind 1,2-DAG or their pharmacological mimic, phorbol esters; CC (brown), a sequence with a strong propensity to form a coiled-coil, and LX (gray), a low-complexity sequence, as predicted by UnitProt [50] and/or SMART [51] databases; AL, activation loop Thr (T983), phosphorylated by Pkh1 (and Pkh2); TM, turn motif Thr (T1125), and, HM, hydrophobic motif Ser (S1143), both phosphorylated by TORC2.

Yeast TORC2 is localized at the PM [27,29,35,36] and responds to certain perturbations and stresses by directly phosphorylating two distinct types of protein kinases: Ypk1 (and its paralog Ypk2/Ykr2) [37] (Figure 1); and, Pkc1 [38] (Figure 2).

TORC2 phosphorylates Ypk1 at multiple sites at its C-terminal end, thereby stimulating its activity [22,24,39]. Ypk1 is a member of the sub-family of eukaryotic protein kinases first defined by the related protein kinases cyclic-3’5’-AMP-dependent protein kinase (PKA), cyclic-3’5’-GMP-dependent protein kinase (PKG), and the conventional Ca^2+^-, DAG-, and PtdSer-activated protein kinases (PKC) (AGC-family of protein kinases) [40,41]. Several criteria indicate that the human ortholog of Ypk1 is SGK1. The catalytic domain of Ypk1 shares a greater degree of amino acid sequence identity (57%) with that of SGK1 than any other human protein kinase [42,43,44]. In addition, the optimal site for phosphorylation of substrates (phospho-acceptor site specificity) of Ypk1 and SGK1 are virtually identical [45]. Moreover, expression of SGK1 (and no other, even closely related, mammalian protein kinase) rescues the inviability of a *ypk1∆ ypk2∆* double mutant [45]. Lastly, as a prerequisite for its TORC2 stimulation, basal Ypk1 activity requires phosphorylation of its activation loop (T-loop) by the PM-localized protein kinases Pkh1 and Pkh2 [46,47]. Pkh1 and Pkh2 are the yeast sequence homologs of the mammalian, PM-associated, 3-phosphoinositide-dependent protein kinase 1 (PDK1) and expression of PDK1 rescues the inviability of a *pkh1∆ pkh2∆* double mutant [45]. As for Ypk1, full SGK1 activity requires its phosphorylation by both PDK1 [48] and mTORC2 [49].

For SGK1, there is some evidence that modification at one of its C-terminal mTORC2 sites, designated its “hydrophobic motif” (FLGF**S**YAPP) obligatorily precedes PDK1-mediated phosphorylation of its activation loop [53], rather than being independent events as we have observed for Ypk1 in yeast [22,39,47]. The crystal structure of PDK1 [54] suggested that this requisite order might arise from the fact that the N-terminal lobe of the PDK1 kinase domain contains a hydrophobic groove (“PIF pocket”) lined with nearby basic (Lys76 and Arg131) and polar (Thr148 and Gln150) side chains capable of coordinating a negatively-charged phosphate group. Hence, it was proposed that, once phosphorylated, the hydrophobic motif of an AGC kinase docks in the PIF pocket, thereby recruiting it to PDK1. Such an intermolecular interaction would then allow PDK1 to phosphorylate the activation loop of the bound AGC kinase (a proximity effect) more efficiently. On the contrary, analysis of crystal structures for the prominent AGC kinase family member AKT1/PKB suggested a different mechanistic explanation for why hydrophobic motif phosphorylation precedes activation loop phosphorylation. These studies showed that AKT1 also possesses a PIF pocket and, when its hydrophobic motif (FPQF**S**YSAS) is phosphorylated, it can dock in the PIF pocket in an intramolecular fashion [55]. This interaction causes a disorder-to-order transition that restructures important elements of the kinase domain, including stabilizing the αC helix and reconfiguring the activation loop [55]. This dramatic conformational change of the activation loop facilitates its recognition and phosphorylation by PDK1 (an accessibility effect). In any event, for Ypk1, phosphorylation of its hydrophobic motif (FGGW**T**^662^YVGN) cannot be an obligatory prelude to Pkh1-dependent phosphorylation of its activation loop based on the following findings. A Ypk1(T504A) mutant, which cannot be and is not phosphorylated on its activation loop [52], is non-functional in vivo because it was unable to support growth, whereas a Ypk1(T662A) mutant supported cell viability as efficiently as wild-type (WT) Ypk1 under standard growth conditions, i.e., on rich medium at 30 °C [47]. Moreover, purified Ypk1(T504A) was catalytically inactive in vitro [45], whereas Ypk1(T662A) was only slightly less active than normal Ypk1 [47]. Nonetheless, when isolated from yeast cells, a phosphomimetic allele, Ypk1(T662D), reproducibly exhibited a two-fold higher specific activity, as compared to WT Ypk1 or Ypk1(T662A) [47]. Hence, it is possible that Pkh1 possesses the equivalent of a PIF pocket and the negative charge in the hydrophobic motif in Ypk1(T662D) mimics phosphate, permitting higher affinity recruitment of Pkh1 and more efficient activation loop phosphorylation. In fact, all of the residues in mammalian PDK1 that constitute its PIF pocket (including the phosphate-binding residues) are conserved in yeast Pkh1 and Pkh2 [47,56]. Alternatively, however, like AKT1, Ypk1 may itself contain a PIF pocket and, when it is occupied by the phosphorylated hydrophobic motif, or the phospho-mimetic version in Ypk1(T662D), it reinforces a conformation that is intrinsically more catalytically active. We favor this latter model, as discussed further below.

The *PKC1* gene also has an apparent human ortholog. *PKC1* was so designated, at the time, because of its resemblance to members of another class of AGC-family protein kinase, the “conventional” mammalian PKC family [38]. However, this classification is a misnomer. Based on its overall organization (Figure 2), the degree of sequence similarity of its catalytic domain to mammalian counterparts, and its biochemical properties in vitro, especially its activation by binding of GTP-bound Rho1 [57], it is now clear that Pkc1 is more closely related to the three mammalian Rho- (and Rac-) activated so-called PKC-related protein kinases, especially PKC-related protein kinase-2 (originally called PRK2, but now PKN2), which are well-documented Rho-GTP-activated protein kinases [58,59,60].

## 4. Structure, Function, and Regulation of Ypk1 

The *YPK1* gene was first identified and isolated via its ability to hybridize to the complementary DNA (cDNA) for a catalytic subunit of bovine PKA [61], a founding member of the AGC-family of protein kinases [40,41]. Likewise, a very highly related gene, initially designated *YKR2* (now called *YPK2*), was cloned via its hybridization to a cDNA for an isoform of rabbit PKC [62], another prototypical AGC kinase. Similarly, both *YPK1* and *YPK2* were independently recovered using hybridization probes derived from cDNAs encoding isozymes of rat PKCs and shown by genetic analysis to be a pair of functionally redundant loci essential for the growth of yeast cells [37]. With determination of the complete DNA sequence of the *S. cerevisiae* genome in 1996–97 [63,64], and now the entire genomes of many related and more distant yeasts [65,66], it is clear that *YPK1* and *YPK2* are true paralogs and together represent one of the distinct classes of AGC kinases present in the fungal clade [42,43,44].

Ypk1 is a 680-residue polypeptide with its catalytic domain located within its carboxy-terminal portion, preceded by a long amino-terminal segment and followed by a much shorter C-terminal extension (Figure 1). Ypk2 (677 residues) shows extensive homology to Ypk1 across its entire length and their catalytic domains share 90% sequence identity (Figure 1 legend). Aside from its homology to that in Ypk2, the N-terminal portion of Ypk1 does not exhibit detectable similarity to any characterized sequence motif or structural domain. By contrast, the C-terminal extension shares readily detectable relatedness (30% identity) to that in SGK1. To date, no regulators of Ypk1 (or Ypk2) that act via direct interaction with the N-terminal segment have been identified. However, several observations indicate that the N-terminal domain exerts some negative regulatory constraint on the C-terminal catalytic domain. First, unlike overexpression of full-length Ypk1, overexpressing an allele that lacks its entire N-terminal domain, Ypk1(∆2-336) (also known as Ypk1∆N), was toxic, whereas overexpressing a kinase-dead (K376A) derivative of Ypk1∆N was not [46], implying that the toxicity arises from the hyperactivity of Ypk1∆N. Consistent with that model, the toxicity of Ypk1∆N was also ameliorated in a *pkh1∆* mutant, which cripples its activation loop phosphorylation [46]. Second, a Ypk1 mutant with a single N-terminal substitution mutation (D242A) rescues the inviability of a *tor2^ts^* allele [22], in agreement with the fact that Ypk1 is a primary TORC2 target, but also suggesting that this alteration of the N-terminal domain alleviates the need for TORC2-dependent phosphorylation of Ypk1. Similarly, it was observed that a corresponding mutation in Ypk2 (D239A) [67] suppressed the growth of cells carrying the *avo3-30* mutation, a temperature-sensitive allele that also compromises TORC2 function at the restrictive temperature [68]. These observations suggest first that, normally, the N-terminal domain interacts with and constrains the kinase fold of Ypk1 in an inactive conformation. These findings further suggest that the role of the C-terminal regulatory segment, when phosphorylated by TORC2, is to compete with and displace this N-terminal inhibitory domain (perhaps by the conformational changes induced by binding of the phosphorylated hydrophobic motif to a PIF-like pocket). If so, then the simplest model to explain the effect of the D242A mutation is that it is sufficient, by itself, to prevent the inhibitory action of the N-terminal domain. 

### 4.1. Activation Loop Phosphorylation by Pkh1 and Pkh2

**Figure 3:**
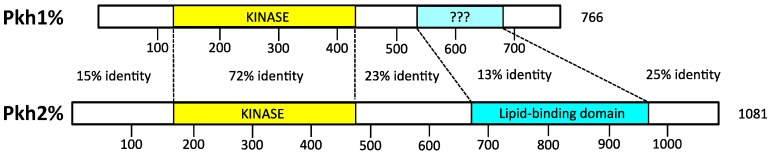
Comparison of Pkh1 and Pkh2. Catalytic domain (yellow), N- and C-terminal extensions (white) and position of a putative lipid-binding domain (blue) characterized in Pkh2 only [70,71,72] are only indicated.

As observed for other AGC family protein kinases [40,41], activation of Ypk1 is regulated by phosphorylation on residues situated within three conserved sequences. First, for any activity at all, Ypk1 must be phosphorylated on its activation loop (T-loop) at Thr504 within a conserved **T**^504^FCGTPEY motif, a modification installed, as mentioned above, by Pkh1 and Pkh2 [46,47]. Pkh1 (766 residues) and the significantly larger Pkh2 (1081 residues) are only closely related within their catalytic domains (72% identity) (Figure 3). So-called Pkh3, which was isolated as a high-copy-number (dosage) suppressor of the temperature-sensitive growth defect of a *pkh1(D398G)^ts^ pkh2∆* double mutant [69], is intermediate in size (898 residues), has a different organization (its catalytic domain is situated at its immediate N-terminal end), and bears substantially less relatedness (46% identity) to either Pkh1 or Pkh2 and only in its kinase domain. Moreover, unlike the inviability of *pkh1∆ pkh2∆* cells, a *pkh3∆* single mutant and *pkh1Δ pkh3Δ* and *pkh2Δ pkh3Δ* double mutants grew comparably to WT cells under all conditions tested [69]. Furthermore, high-level expression of *PKH3* was unable to rescue the inviability of *pkh1∆ pkh2∆* cells [69], indicating, first, that the *pkh1(D398G)^ts^* allele must retain some physiological function in the cell even at a restrictive temperature and, second, that Pkh3 cannot truly compensate for the lack of Pkh1 and Pkh2. 

Pkh1 and Pkh2 are tethered to the PM via their tight association with Pil1, a phosphatidylinositol-4,5-*bis*phosphate (PtdIns4,5P2)-binding protein containing an N-terminal region of homology to the human proteins BIN1 and Amphiphysin and the yeast protein Rvs167 (BAR domain) that is most similar to that in human proteins FER and CIP4 (F-BAR domain) [73], and its paralog Lsp1 [74], which are primary subunits of peripherally PM-bound protein complexes, dubbed eisosomes [75,76], that bear certain similarities to mammalian caveoli [77]. In fact, the interaction between Pkh1 and Pil1 is so robust that it was detected in two independent genome-wide two-hybrid screens [78,79], as well as in two independent genome-wide analyses of isolated tandem affinity purification (TAP)-tagged protein complexes by mass spectrometry (MS) [80,81]; and, in our hands, Pkh1 and Pil1 can be readily and reciprocally co-immunoprecipitated [82]. Initially, eisosomes were thought to be special sites of endocytosis [75], distinct from the clathrin- and actin-dependent route [83], but further analysis indicated that eisosomes are not sites for endocytic entry [84]. Given that *pil1∆* mutants lack normal eisosomes and that even *pil1∆ lsp1∆* double mutants are viable [75,85], the exact role of these structures in cell physiology has remained elusive. Because Pil1 and Lsp1 binding causes the PM to invaginate, it has been suggested that these structures serve as reservoirs of “extra” membrane material on which the cell can draw when it needs to expand rapidly, e.g., under hypotonic conditions [86]. It has also been suggested that, aside from PtdIns4,5P2 binding by Pil1 and Lsp1, eisosomes have another function in maintaining the level of PtdIns4,5P2 in the inner leaflet of the PM because it has been observed that Inp51, one of three synaptojanin-like PtdIns4,5P2 5-phosphatases in *S. cerevisiae* [87,88], specifically associates with Pil1, thereby recruiting this enzyme to the PM [89]. On the other hand, it has been reported previously that, instead, Inp51 is recruited to the PM via specific interaction with Tax4 (and its paralog Irs4), which are orthologs of mammalian EPS15 [90]. Similarly, in *S. pombe*, there is evidence that eisosomes are involved in regulating PM PtdIns4,5P2 levels [91] and localization [92].

In addition to their interaction with Pil1, it has been reported that both Pkh1 and Pkh2 are able to bind directly in vitro to two distinctly different types of negatively-charged lipids, PtdIns4,5P2 and the phosphorylated form of the sphingoid long-chain base dihydrosphingosine-1-phosphate (DHS-P) [70] and, further, that these interactions are synergistic [93]. If the cooperative effect of these two lipids is crucial for either the localization or function of Pkh1 and/or Pkh2, and thus for their ability to activate their downstream targets, it may explain, at least in part, why production of PtdIns4,5P2 is essential for yeast cell growth [94] as well as reports that DHS-P production is needed for maximal growth rate and for survival in response to heat stress [95,96].

In this same regard, it was reported some time ago that Pkh1 and Pkh2 can be stimulated by the long-chain base PHS in vitro [97], and another group claimed to see the same effect [98,99], raising speculation that PHS (or a derived product) serves as a “second messenger” emanating from the sphinogolipid pathway that is critical for Pkh1- and Pkh2-mediated Ypk1 activation [100,101]. However, using a phosphosite-specific antibody that we demonstrated only recognizes Ypk1 phosphorylated at its Pkh1- and Pkh2-dependent site (T504) [45,52], we found that neither blocking production of endogenous PHS (by myriocin treatment) nor elevating intracellular PHS (by supplying copious exogenous PHS) had any effect on the level of T504 phosphorylation in vivo [52]. Thus, there is no credible, physiologically relevant evidence that, in the cell, activation loop phosphorylation of Ypk1 by Pkh1 and Pkh2 is controlled by PHS or any derived product. Thus, the conclusion that Pkh1 and Pkh2 are “sphingolipid-regulated” protein kinases, a concept which seems to continue to be propagated in the literature [70,102], is not correct.

In any event, using both a lipid overlay assay and a flotation assay to assess binding to liposomes, the putative PtdIns4,5P2-binding element in Pkh2 has been localized to a ~300-residue “PH domain-like” segment of its C-terminal extension [71,72]. Curiously, however, despite the reported similarity in lipid-binding properties of Pkh1 and Pkh2 [70], there is very little sequence relatedness between the purported lipid-binding element in Pkh2 and the corresponding region in Pkh1 (Figure 3). Two transmembrane proteins that interact with each other, Sng1 and Nce102, the latter of which localizes to eisosomes, reportedly also contribute to optimal Pkh1 and Pkh2 signaling [103,104], but the mechanism by which they do so is unclear.

**Figure 4:**
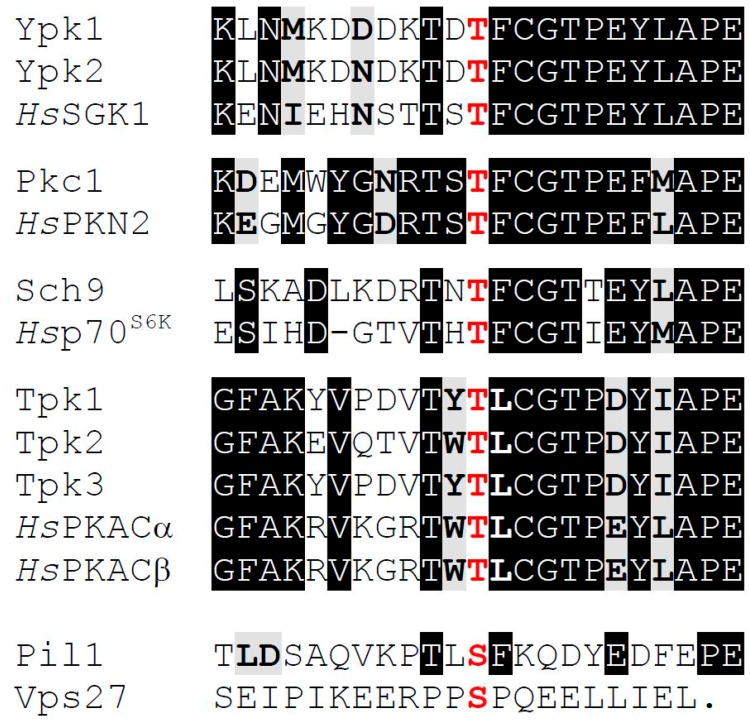
Sites phosphorylated by Pkh1 and Pkh2. The activation loop sequence in the indicated *S. cerevisiae* protein kinases (phosphorylated by Pkh1 and Pkh2) and their corresponding human counterparts (phosphorylated by PDK1), a putative Pkh1 site in the fungal-specific protein Pil1, and a purported Pkh1 site in the ESCRT-0 subunit Vps27 were aligned by anchoring on the residue phosphorylated (bold red). Identities (white letters on black boxes) and standard conservative substitutions (bold letters on grey boxes), and a one-residue gap (hyphen) to maximize one alignment, are indicated. Period (.) indicates the end of the open-reading-frame. Sequence sources were the Saccharomyces Genome Database (SGD; http://www.yeastgenome.org; Stanford University, CA, USA) and the Proteome™ Database of geneXplain GmbH (https://portal.genexplain.com/cgi-bin/portal/login.cgi; Wolfenbüttel, Germany).

Given the tight association of Pkh1 and Pkh2 with eisosomes, all of the target AGC protein kinases that they phosphorylate—including Pkc1 [47,69]; Ypk1 and Ypk2 [46,47,98]; Sch9 [47,56,98]; and Tpk1, Tpk2, and Tpk3 [105] (Figure 4)—must each have some mechanism to bring them to the PM for their encounter with these upstream activators. Before it was appreciated that Pil1 and Lsp1 were the primary components of eisosomes, it was claimed that they served as negative regulators of Pkh1 and Pkh2 activity [85]; however, these conclusions were based on Ypk1 mobility shift assays that were presumed to be due to its Pkh1-mediated phosphorylation, but were later shown to be due to mobility shifts arising from phosphorylation of Ypk1 at sites phosphorylated by the protein kinases Fpk1 and Fpk2 [52] (Figure 1), which are discussed further below. Pil1 itself is, however, an apparent substrate of Pkh1, and its purported Pkh1-dependent modification has some influence on eisosome assembly and organization [85,99,106]. Surprisingly, however, the sequence of even the most well-characterized of the reported Pkh1 sites in Pil1 bears only very modest resemblance to the rather strikingly conserved sequence context in which the Thr of the activation loop is embedded in all of the AGC protein kinases that are Pkh1 and Pkh2 targets (Figure 4). Aside from Pil1, there are also reports that Pkh1 can phosphorylate at least two other substrates. It is claimed that Pkh1 can bind to and phosphorylate Gcn2 (the yeast eIF2α kinase) in vitro, although the putative site(s) modified were not identified and inactivation of Pkh1 function had no effect in vivo on any readout of Gcn2 function [107]. It has also been claimed that Pkh1 phosphorylates Vps27 [108], a component of the Endosomal Sorting Complex Required for Transport (ESCRT) pathway. Vps27 (a VHS-, FYVE-, and UIM-domain containing protein) is one of the two subunits of the cargo recognition complex (ESCRT-0) that initiates and recruits the other downstream ESCRT complexes [109,110]. The putative Pkh1 site in Vps27 is S613, but, this residue lies in a sequence context that bears no discernible similarity to the T-loops of the AGC kinase targets of Pkh1 or even the site in Pil1 (Figure 4), and the evidence that Pkh1 is the protein kinase responsible for installing this modification in vivo is unconvincing.

### 4.2. C-Terminal Phosphorylation by TORC2

**Figure 5:**
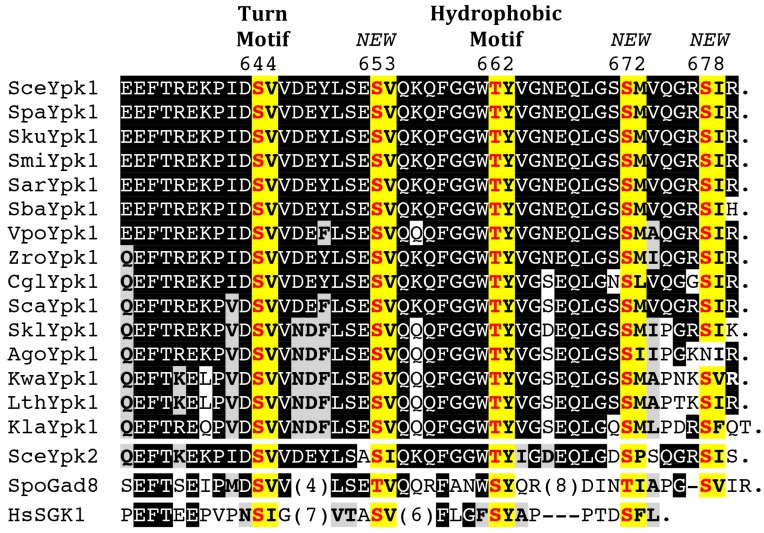
Conservation of sites phosphorylated by TORC2 in Ypk1. The C-terminal end of *Saccharomyces cerevisiae* (Sce) Ypk1 (top line) was aligned with the corresponding segment of Ypk1 orthologs from sensu stricto Saccharomyces species *S. paradoxus* (Spa), *S. kudriavzevii* (Sku), *S. mikatae* (Smi), *S. arboricola* (Sar), and *S. bayanus* (Sba), more divergent species *Vanderwaltozyma polyspora* (Vpo), *Zygosaccharomyces rouxii* (Zro), *Candida glabrata* (Cgl), *S. castellii* (Sca), *S. kluyveri* (Skl), *Ashbya gossypii* (Ago), *Kluveromyces waltii* (Kwa), *Lachancea thermotolerans* (Lth), and *Kluveromyces lactis* (Kla), SceYpk2, *Schizosaccharomyces pombe* (Spo), and *Homo sapiens* (Hs) SGK1 (bottom line). Identities (white letters on black boxe*s*) and standard conservative substitutions (bold letters on grey boxes) shared between SceYpk1 and another ortholog are indicated. One-residue gaps (hyphens) and insertions of the indicated length (parentheses) were introduced to maximize alignment of the most distant orthologs. Period (.) indicates the end of the open-reading-frame. The proximal seven-residue insert in HsSGK1 contains two additional potential mTORC2 sites (KSPDSVL). Sequence sources can be found in [39].

Pkh1- and Pkh2-mediated phosphorylation of the activation loop in Ypk1 and Ypk2 is sufficient to confer basal activity. However, for cell survival in response to certain stresses (e.g., sphingolipid depletion, heat shock, hypotonic conditions, high exogenous acetic acid), Ypk1 activity must be upregulated further by phosphorylation at Thr662 within its conserved hydrophobic motif sequence near its C-terminus [22,28,29,30,47,67,111,112] (Figure 1 and Figure 5). As first revealed by analysis of Ypk2 [67], phosphorylation at the hydrophobic motif is mediated by TORC2, which also phosphorylates another C-terminal site (Ser644 in Ypk1) within another conserved sequence, dubbed the turn motif [47,67] (Figure 5). Crystal structures of mammalian AGC kinases have revealed that, when phosphorylated, the hydrophobic motif docks in the PIF pocket in the N-lobe of the kinase domain [55,113] and, likewise, when phosphorylated, the turn motif interacts with another positively-charged pocket in the N-lobe [114,115]. In both cases, these interactions induce restructuring of the kinase domain, stabilizing it in the conformation found in the fully active state.

Recently, we have shown that several additional sites in Ypk1, aside from the turn and hydrophobic motif, are also phosphorylated by TORC2 (Figure 5) and that these sites are as important for Ypk1 activity, stability, and biological function as Ser644 and Thr662 [39]. Moreover, phosphorylation at these sites is a prerequisite for TORC2 phosphorylation of Thr622 in the hydrophobic motif [39]. Quite similarly, the mammalian AGC kinase AKT1 is phosphorylated in an mTORC2-dependent manner at other C-terminal sites that are necessary for efficient phosphorylation of its hydrophobic motif [116]. Furthermore, phosphorylation at the turn motif has been shown to be especially important for proper carboxyl-terminal folding and stability of mammalian AKT and PKC [117,118]. On this basis, it seems likely that phosphorylation of the newly identified TORC2 sites in Ypk1 is also a prelude to phosphorylation of Ser644 in its turn motif because absence of phosphorylation at the new sites markedly reduces Ypk1 stability. These observations make it clear that TORC2 plays a key role in stimulating Ypk1 activity, up to the level needed by the cell to cope with the stresses of sphingolipid depletion, heat shock, hypotonicity, and other stressful conditions known to perturb PM structure and/or function.

There has been some degree of controversy about how TORC2 recognizes Ypk1 (and Ypk2) as substrates. Two groups have reported [28,29] that Ypk1 is delivered to the catalytic core (Tor2-Lst8) of TORC2 via its direct physical association with an ancillary subunit, Slm1 (and its paralog Slm2) [119,120]. In marked contrast, in both animal cells [121,122] and fission yeast [123,124], there is compelling evidence that the Avo1 orthologs (mSIN1 and Sin1, respectively) in TORC2 bind and are required for phosphorylation of the Ypk1 orthologs in these organisms (SGK1 and Gad8, respectively). Likewise, others have shown that *S. cerevisiae* Ypk2 also seems to interact with TORC2 via binding to Avo1 [125]. Moreover, based on cryo-EM, cross-linking MS, and other approaches, the current model of yeast TORC2 structure [15,21] suggests that Slm1 and Slm2 are peripheral subunits that dock quite far from the catalytic center of the complex (via their interaction with the non-essential subunits Bit61 and/or its paralog Bit2, as well as Avo2 [119,126]). By contrast, the structural models show that Avo1 is located in close proximity to the active site of the Tor2-Lst8 complex. Furthermore, convincing data show that a sequence shared by Avo1 with both *S. pombe* Sin1 and mammalian mSIN1, designated the “conserved region in the middle” (CRIM), is a discrete domain that adopts a stable ubiquitin-like fold with a prominent acidic loop and is necessary and sufficient for binding of Gad8 and SGK1, respectively [127]. For example, CRIM^Sin1^ fused to a different TORC2 subunit permits Gad8 hydrophobic motif phosphorylation in an *S. pombe sin1∆* mutant. Therefore, it is likely that Ypk1 is recognized by TORC2 by binding to the CRIM element in Avo1.

However, it is incontrovertible that, normally, Slm1 and Slm2 are required for TORC2 function, including Ypk1 phosphorylation at its C-terminal sites [26,28,29]. So, some essential role, other than substrate delivery, needs to be invoked to explain how these proteins contribute to Ypk1 stimulation. Aside from its CRIM domain, Avo1, which is tightly Tor2-bound, has a C-terminal PtdIns4,5P2-specific PH domain [70,93] that is reportedly necessary for efficient PM localization of TORC2 [27]. Likewise, both Slm1 (and Slm2) have C-terminal PH domains [128,129], which have been shown to be specific for binding PtdIns4,5P2 [70,93,128]. It is possible, therefore, that Slm1 and Slm2 help reinforce stable PM recruitment of TORC2 and its positioning near Pkh1- and Pkh2-bound eisosomes, which, as discussed above, are located at PM sites enriched for PtdIns4,5P2. Alternatively, or in addition, Slm1 and Slm2 might help localize TORC2 nearby ER-PM junctions, which also control phosphoinositide metabolism and vice-versa [130]; in fact, several of the demonstrated substrates of Ypk1 are endoplasmic reticulum (ER)-localized proteins, as discussed below. In any event, the level of PtdIns4,5P2 is indeed very important for TORC2 function because it was demonstrated quite some time ago that elevating PM PtdIns4,5P2 levels either by deleting the PM-localized synaptojanin Inp51 or overproducing the PM-localized PtdIns4P 5-kinase Mss4 rescued the temperature-sensitive lethality of the *tor2-21^ts^* allele [90].

It is also noteworthy that one way Slm1 and Slm2 were identified initially is through their capacity to bind calcineurin (phosphoprotein phosphatase 2B) very tightly [120,131,132]. Moreover, essentially all the subunits of both mammalian and yeast TORC2 are demonstrated phospho-proteins ([21]; see also data available at the Saccharomyces Genome Database). If any such modifications, alone or cumulatively, are inhibitory to TORC2 function, it is possible that the important role of Slm1 and Slm2 is to facilitate the calcineurin-mediated reversal of such negative regulation. In this same regard, it is of some interest that, other than the AGC family protein kinases, the only other direct substrate of TORC2 for which there is any evidence is Slm1; reportedly, Slm1 is phosphorylated in a TORC2-dependent manner in vivo and immunoprecipitated Tor2 (but not a kinase-dead variant) phosphorylated Slm1 and Slm2 in vitro [133]. However, the sites of phosphorylation were not mapped and, thus, no mutations were made to determine whether there are any detectable physiological consequences to the presence or lack of these modifications. 

Whatever the essential roles of Slm1 and Slm2 in TORC2-Ypk1 signaling, their interaction with TORC2 cannot be obligatorily through association with Bit61 (and/or Bit2) because, unlike *slm1∆ slm2∆* double mutant cells, *bit2∆ bit61∆* double mutant cells are viable [134]. Similarly, in the mouse, *protor-1 (prr5)^-/-^ protor-2 (prr5L)^-/-^* nullizygous animals are viable and, for as yet unknown reasons, the only tissue from such Protor-deficient animals in which efficient SGK1 activation seems impaired is the kidney [135].

### 4.3. N-Terminal Phosphorylation by Fpk1 and Fpk2

When examined by standard SDS-PAGE, especially under conditions known to improve the resolution of phospho-isoforms (75:1 or 100:1 acrylamide: *N,N′*-methylene-*bis*-acrylamide), Ypk1 migrates as a set of three or four distinct bands; however, none of these species could be attributed to its Pkh1- or TORC2-dependent modification, based on mutation of the corresponding sites or on inactivation of the cognate protein kinases [52]. A genome-wide screen of a protein kinase mutant collection revealed that the protein kinases responsible for the observed Ypk1 mobility shifts are Fpk1 and its paralog Fpk2/Kin82 [52]. Fpk1 phosphorylates Ser51 and Ser71 within a defined sequence context (-R-x-S>T-**L/V/I/M/A**-D/E-) [26,52,136] in the N-terminal regulatory domain of Ypk1 (Figure 1).

**Figure 6:**
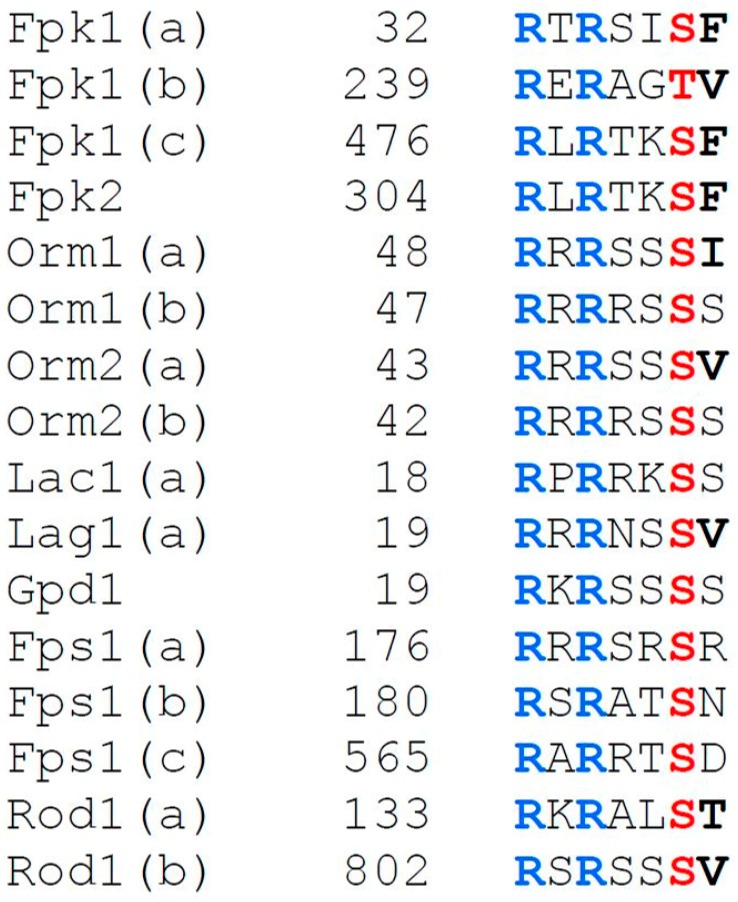
Selected sites of Ypk1 phosphorylation. The phospho-acceptor sites in the indicated gene products (position of first residue in the sequence shown indicated on the left) were validated as authentic Ypk1 phosphorylation sites both in vitro and in vivo. Documentation for these assignments can be found in the following publications: Fpk1 and Fpk2 [52]; Orm1 and Orm2 [22]; Lac1 and Lag1 [23]; Gpd1 [137]; Fps1 [24]; and, Rod1 [25].

Strikingly, Fpk1 is itself a substrate of Ypk1. Ypk1 also has a well-defined phosphoacceptor site specificity [-R-x-R-x-x-S>T-(Hpo)-, where (Hpo) indicates only a modest preference for a hydrophobic residue] [45,136]. Fpk1 (893 residues) possesses three consensus Ypk1 phosphorylation motifs in its N-terminal regulatory domain (Figure 6) and the significantly shorter Fpk2 (720 residues) has one (Figure 6). Ypk1-mediated phosphorylation of Fpk1 and Fpk2 inhibits their function [52], which, in turns, blocks their ability to phosphorylate Ypk1. Thus, when cells are treated with a stimulus that activates TORC2-Ypk1 signaling, Ypk1-dependent phosphorylation of Fpk1 increases, resulting in reduced Fpk1-mediated phosphorylation of Ypk1, eliminating the slower mobility isoforms of Ypk1 observed in standard SDS-PAGE. Thus, there is an interesting reciprocal relationship between these two classes of protein kinases; and, the extent of these Ypk1 mobility shifts serves as a convenient in vivo read-out of Fpk1 (and Fpk2) activity. In this regard, Fpk1 activity in vivo depends, not only on escape from Ypk1-mediated inhibition, but also on a complex sphingolipid, namely mannosyl-inositolphosphoryl-ceramide (MIPC) [26,52], although whether MIPC is a direct allosteric activator or acts via a more indirect mechanism has not been elucidated.

In contrast to the clear-cut experimental evidence both in vitro and in vivo that Ypk1-mediated phosphorylation of Fpk1 inhibits its catalytic function, the effect of Fpk1-mediated phosphorylation on Ypk1 has been more difficult to discern [52]. In *S. pombe*, N-terminal phosphorylation of Gad8 (an ortholog of *S. cerevisiae* Ypk1) on Thr6 by Pck2 (an ortholog of *S. cerevisiae* Pkc1) reportedly prevents physical association of Gad8 with TORC2, decreases TORC2-mediated phosphorylation of its hydrophobic motif (Ser546), thus reducing TORC2-mediated activation of Gad8 [138]. Hence, it is tempting to speculate that, in the same way, Fpk1-mediated phosphorylation of Ypk1 is a negative feedback mechanism to dampen TORC2-mediated activation of Ypk1. Such a logic circuit would make physiological sense because, as discussed further below, one of the primary functions of TORC2-Ypk1 signaling is to upregulate the production of complex sphingolipids. When the supply of cellular sphingolipids is adequate, Ypk1 action is no longer needed for that purpose. Given that optimal Fpk1 function requires cellular MIPC production, its phosphorylation of Ypk1 likely serves as a rheostat to adjust the level of Ypk1 activity to meet the demands of the cell for sphingolipids in a finely tuned manner.

### 4.4. Ypk1 Activity Is Not Controlled by Sterol 

In marked contrast to its activation upon sphingolipid depletion [22,28], TORC2 does not appear to serve as a sensor of sterol stress, based on several observations. TORC2 is not activated when sterol synthesis is blocked by treatment of cells with lovastatin [139], a potent inhibitor of HMG-CoA reductase, a key enzyme in the mevalonate pathway, that blocks isoprenoid and sterol synthesis in yeast [140,141,142]. In a similar regard, on the basis of a proteome-wide lipidomics screen, it was reported that Ypk1 binds ergosterol and that Ypk1 activity in vitro is stimulated by ergosterol [143], suggesting that Ypk1 itself might be a direct sterol sensor. However, subsequent work documented that, in the cell, absence of ergosterol does not detectably diminish either basal or myriocin-induced Ypk1 activity [22]. 

## 5. Substrates of Ypk1

### 5.1. Control of Aminoglycerophospholipid Asymmetry in the Plasma Membrane Bilayer 

The first bona fide targets of Ypk1 discovered were Fpk1 and Fpk2. They were likely candidates for several reasons. First, Ypk1- and Ypk2-deficient cells display defects in PM-associated functions [32,46,111]. Second, Fpk1 was shown to phosphorylate and thereby stimulate at least two PM-localized aminoglycerophospholipid flippases [144]. Flippases are P-type ATPases (class IV) that translocate lipid clients from the outer leaflet to the inner leaflet of the PM bilayer [145]. Third, in a screen for transposon insertion mutations that suppressed the temperature-sensitive growth defect of *ypk1^ts^ ypk2Δ* cells, one insertion that was isolated disrupted the coding sequence for a flippase [46], suggesting that one role of Ypk1 is to negatively regulate flippase function, either directly or indirectly. Finally, as already mentioned above, Fpk1 has three consensus Ypk1 phosphorylation motifs in its N-terminal regulatory domain and its minor paralog Fpk2 has one (Figure 6). Indeed, it was demonstrated that Fpk1 (and Fpk2) are phosphorylated robustly by Ypk1 in vitro and at the predicted sites and, likewise, that Fpk1 is phosphorylated in a Ypk1-dependent manner in vivo at the expected sites [52]. Moreover, it was shown that an Fpk1(S37A T244A S481A) mutant that is immune to Ypk1 phosphorylation is hyperactive both in vitro and in vivo and, conversely, that the absence of Fpk1 and Fpk2 suppresses the temperature-sensitive growth of *ypk1^ts^ ypk2Δ* cells [52]. These results and other more recent findings [26] support the conclusion that TORC2-Ypk1 signaling down regulates the rate of inward aminoglycerophospholipid translocation (mainly PtdEth) by inhibiting the ability of Fpk1 (and Fpk2) to phosphorylate and thereby stimulate the flippases. Moreover, based on the observation that Fpk1(S37A T244A S481A) activity was prevented when sphingolipid synthesis was blocked, revealed that there was a separate Ypk1-independent mechanism for controlling Fpk1 function, which was traced to the need for MIPC [52], as was already discussed in a preceding section. Overall, this regulatory circuit, with its interplay between Ypk1 and Fpk1, provides a mechanism to couple the rate of aminophospholipid flipping within the PM to the rate of synthesis of a complex sphingolipid. The more MIPC available, the faster the internalization of outer leaflet PtdEth. Maintaining this balance in PM lipid composition and leaflet distribution appears to be crucial for many aspects of membrane function because defects in flippase activity have been reported to cause problems in the vesicle-mediated transport of proteins in both the endocytic and exocytic pathways [146], in the non-vesicular trafficking of sterols [147], and in maintaining the axis of polarized growth through effects on PM recruitment of Cdc42 [148,149]. Because counterparts of TORC2, Ypk1, and Fpk1 are found throughout phylogeny (although TORC2 core proteins seem to be missing in photosynthetic organisms [150]), it is possible that analogous regulation occurs in mammals. Indeed, proper distribution of PtdEth (and PtdSer) between the outer and inner leaflets of the plasma membrane in animal cells is necessary for membrane protein activity, for vesicle biogenesis, and for cell signaling, morphology, and movement [151]. Elevated levels of aminoglycerophospholipids (especially PtdSer) in the extracellular leaflet can initiate many responses, including phagocytosis, platelet activation, and apoptotic death [151,152,153].

### 5.2. Control of Sphingolipid Biosynthesis 

The first clue that Ypk1 action was centrally involved in regulating sphingolipid production came from the fact that *SLI2*, one of the genes isolated in a screen for dosage suppressors of “SphingoLipid inhibition by ISP-1”, was isogenic to *YPK1* [154]. It had been established previously that the antibiotic ISP-1, more commonly known as myriocin [155,156,157], inhibits eukaryotic cell growth because it is a transition state mimic that potently blocks l-serine:palmitoyl-CoA C-palmitoyltransferase (decarboxylating) (SPT; Lcb1-Lcb2-Tsc3 heterotrimer in yeast), the first enzyme unique to the sphingolipid biosynthetic pathway in all eukaryotes [101,158,159,160]. Overproduction of a Ypk1 mutant lacking catalytic activity did not suppress growth inhibition by myriocin. These findings suggested that elevating the amount of Ypk1 activity was able to somehow overcome the limitation for sphingolipids caused by reducing the rate of their synthesis with the inhibitor; however, no targets of Ypk1 were identified. The next evidence that a protein kinase downstream of TORC2 was important for controlling the rate of sphingolipid production came from the observations, first, that an *avo3-30^ts^* mutation, which cripples TORC2 function at the non-permissive temperature, also greatly diminished the amount of ceramides in the cell under the same conditions and, second, that expression of the hyperactive D239A allele of the Ypk1 paralog Ypk2 was able to suppress the growth defect of *avo3-30^ts^* cells at the restrictive temperature [68]. It had already been established that Ypk2 was a downstream target of TORC2 [67]. These findings suggested that sphingolipid depletion stimulated TORC2-dependent phosphorylation of Ypk2, which, in turn, somehow promoted more sphingolipid production; however, no direct substrate of Ypk2 was pinpointed.

In 2002, we carried out a comprehensive screen for both dosage (gain-of-function) suppressors and transposon insertion (loss-of-function) suppressors of the temperature-sensitive lethality of a *ypk1^ts^ ypk2∆* strain [46]. The former potentially represent substrates whose function requires stimulation by Ypk1-mediated phosphorylation and the latter potentially represent substrates whose function requires inhibition by Ypk1-mediated phosphorylation. One of the best suppressors was a transposon insertion in open-reading-frame YLR350w (now designated *ORM2*). However, none of the suppressors were able to rescue the inviability of a *ypk1∆ ypk2∆* double mutant, emphasizing the importance of the residual Ypk1 activity in the *ypk1^ts^ ypk2∆* strain. That observation, combined with the fact that no single suppressor or combination of two different suppressors was able to fully rescue either the *ypk1^ts^ ypk2∆* cells or the *ypk1∆ ypk2∆* mutant, implied that Ypk1 must have multiple targets. At that time, Orm2 and its paralog Orm1 were so-called “pioneer proteins” because nothing was known about them, or their orthologs in other organisms, with regard to their molecular function. However, that picture changed dramatically in 2010, when it was demonstrated that Orm1 and Orm2 are small, ER-localized tetraspanins that physically associate with and act as negative regulators of SPT and that, upon sphingolipid depletion, both Orm1 and Orm2 become heavily phosphorylated, which alleviates their inhibitory effect on SPT [161]. Moreover, Orm2 appears to be the major isoform [162,163]. These findings immediately suggested that Ypk1 is the protein kinase responsible for phosphorylating Orm1 and Orm2 when sphingolipids become limiting. Indeed, we established that myriocin-induced hyperphosphorylation of Orm1 and Orm2 does not occur in *ypk1Δ* cells, that immuno-purified Ypk1 phosphorylated Orm1 and Orm2 robustly in vitro and did so exclusively on the residues that correspond to its myriocin-induced sites [22]. These conclusions were corroborated by others [28]. Furthermore, we found that an *orm1∆ orm2∆* double mutant was a more robust suppression of the temperature-sensitive growth of the *ypk1^ts^ ypk2Δ* cells than the original transposon insertion in *ORM2* alone, confirming that a primary physiological role of Ypk1-mediated phosphorylation is to negatively regulate their function. Moreover, we documented that Ypk1 activation upon sphingolipid depletion required TORC2-mediated phosphorylation of Thr662 in its hydrophobic motif [22]. Further support for the conclusion that regulation of sphingolipid biosynthesis is one of the most physiological important functions under TORC2 control was the findings that viable *tor2∆* spores could be recovered if they also carried *orm1∆ orm2∆* mutations [34]. Thus, when under the stress of sphingolipid limitation, the cell responds by increasing metabolic flux into the sphingolipid biosynthetic pathway through the increase in SPT activity that is brought about via the TORC2-Ypk1-mediated phosphorylation and inhibition of the SPT inhibitors, Orm1 and Orm2.

In a subsequent global screen for additional candidate Ypk1 substrates using a combined bioinformatic, genetic, and biochemical screening procedure [23], we identified the two catalytic subunits (Lac1 and its paralog Lag1) of the ceramide synthase complex, another ER-localized pacemaker enzyme in sphingolipid synthesis, as direct substrates of Ypk1. In this instance, however, phosphorylation by Ypk1 stimulates the function of this enzyme. Furthermore, we showed that the TORC2-Ypk1-driven increase in ceramide synthase activity ensures that the long-chain base precursors made by the TORC2-Ypk1-driven increase in SPT activity are efficiently channeled into the production of complex sphingolipid end-products of the yeast sphingolipid biosynthetic pathway [23]. Exerting control at this step of the pathway also prevents accumulation of pathway intermediates that would otherwise compromise cell growth by stimulating autophagy [164]. 

### 5.3. Control of Intracellular Glycerol Concentration 

In *S. cerevisiae*, glycerol-3-phosphate (Glo3P) derived by NADH-dependent reduction of the glycolytic intermediate dihydroxyacetone-phosphate (DHAP) sits at an important metabolic node. Under normal growth conditions, Glo3P can be esterified at its *sn*-1 and *sn*-2 hydroxyls with fatty acyl groups, catalyzed by Gpt2 and Sct1, to form phosphatidic acid (PtdOH), which, in turn, can be converted to various classes of glycerophospholipids either via Cds1-catalyzed CDP-DAG formation (leading to PtdIns, PtdSer, and PtdGlo) or via Pah1-catalyzed dephosphorylation to DAG (leading to PtdEth and PtdCho via the Kennedy pathway) [165,166]. Alternatively, DAG can be esterified with a fatty acyl group at its *sn*-3 hydroxyl, catalyzed by Dga1 (and Lro1), to generate triacylglycerol that is stored in lipid droplets [167,168,169]. 

However, when subjected to the stress of being placed in hypertonic conditions, Glo3P in the yeast cell has a completely different fate—it is dephosphorylated by two phosphatases (Gpp1/Rhr2 and Gpp2/Hor1) whose cognate genes are upregulated upon hyperosmotic stress [170]. Generation of high internal glycerol is the strategy yeast has evolved to provide a sufficient concentration of an inocuous intracellular osmolyte to combat the loss of water [171]. The effector of a signaling pathway, the HOG (high osmolarity glycerol) response, that controls the processes needed to cope with hyperosmotic stress, including induction of the expression of appropriate genes, is the MAPK Hog1 [172] 

Given the above considerations, it is not surprising that the reduction of DHAP, catalyzed by two paralogous dehydrogenases (Gpd1 and Gpd2) [173], is regulated by mechanisms that allow for a rapid switch between the two distinct metabolic uses for the Glo3P produced. Gpd2, which is located in the cytosol and inside the mitochondrion, is constitutively active, but is inactivated when glucose is limiting via phosphorylation at an N-terminal site by the energy stress-responsive protein kinase Snf1 (mammalian ortholog is AMPK) [137]. Thus, it is ensured that little Glo3P is made under conditions where fatty acids need to be “burned” for energy generation rather than diverted into either phospholipid or triacylglycerol production. In contrast, Gpd1, which is found in the cytosol and inside peroxisomes, is kept inactive via phosphorylation by Ypk1 at a site analogous to that in Gpd2; however, TORC2-Ypk1 signaling is dramatically and rapidly decreased when cells are subjected to hyperosmotic shock [137]. Thus, under conditions were Glo3P is needed for glycerol production, Ypk1-mediated inhibition of Gpd1 function is rapidly alleviated. Furthermore, this effect is potentiated by the fact that, upon hyperosmotic stress, *GPD1* mRNA and protein expression are markedly upregulated in a Hog1-dependent manner [174], allowing the level of Glo3P for glycerol production to ramp up quickly.

However, glycerol will only accumulate inside the cell if a PM-localized channel, the aquaglyceroporin Fps1, closes. A primary mechanism for channel closure involves Hog1-mediated phosphorylation and displacement of its positive regulators, Rgc1 and Rgc2 [175]. However, that effect takes some time and, moreover, Fps1 still closes in response to hyperosmotic shock even in *hog1∆* cells [176,177], indicating another mechanism to prevent Fps1-mediated glycerol efflux. Strikingly, in our proteome-wide screen [23], Fps1 was identified as a likely target of Ypk1. This conclusion was corroborated by showing that Fps1 is an authentic Ypk1 substrate in vitro and in vivo and that the open channel state of Fps1 requires its phosphorylation by Ypk1 at three sites [24]. We further showed that under hyperosmotic conditions, where TORC2-Ypk1 signaling is rapidly decreased, Fps1 phosphorylation is lost, causing channel closure, glycerol accumulation, and enhanced survival under hyperosmotic stress [24]. Thus, inactivation of TORC2-Ypk1 signaling upon hyperosmotic shock has two coordinated consequences that work synergistically to cause glycerol accumulation and promote cell survival, outcomes that work in conjunction with, but in a much more rapid and mechanistically distinct manner from, the processes evoked by activated Hog1. First, in less than one minute, loss of TORC2-Ypk1 signaling alleviates inhibition of Gpd1—which, combined with transcriptional induction of *GPD1*, *GPP1* and *GPP2*—greatly increases the rate of glycerol production. Second, loss of TORC2-Ypk1 signaling also rapidly closes the Fps1 channel, thereby allowing the glycerol produced to be retained. These findings defined the underlying molecular basis of a previously uncharacterized mechanism for responding to hypertonic conditions [24]. Moreover, the fact that a minor sequence change at an analogous phosphorylation site placed two closely-related metabolic enzymes, Gpd1 and Gpd2, under the control of two distinct classes of stress-activated protein kinases (Ypk1 and Snf1, respectively) suggests that phosphorylation site divergence can be a contributory driving force for functional specialization of the products of paralogous genes during evolution.

### 5.4. Control of Integral Plasma Membrane Protein Endocytosis

In addition to modulating the levels and leaflet distribution of glycerophospholipids and the rate of sphingolipid biosynthesis, Ypk1 also influences PM homeostasis by down regulating the rate of endocytosis of integral membrane proteins in at least two ways. Using the α-factor pheromone receptor Ste2 as a model polytopic membrane protein, which is internalized in response to both its constitutive and agonist-induced ubiquitinylation [178], we found that Ypk1-mediated phosphorylation at two sites blocked the ability of the endocytic adaptor (α-arrestin) Rod1/Art4 to mediate ligand-induced internalization of Ste2 [25]. The α-arrestins promote ubiquitinylation of their respective cargo molecules and their engagement by the clathrin-dependent endocytic machinery because these adaptors recruit the membrane-associated HECT-domain ubiquitin ligase Rsp5. The *S. cerevisiae* genome encodes 14 recognized α-arrestins, most of which have been implicated in endocytosis and trafficking of a wide variety of nutrient permeases [179,180,181]. The majority of these adaptors, including Ldb19/Art1 (1), Ecm21/Art2 (4), Aly2/Art3 (3), Ygr068/Art5 (1), Aly1/Art6 (1), Rog3/Art7 (2), Csr2/Art8 (3), Rim8/Art9 (1), and Ylr392c/Art10 (1), may also be negatively regulated in manner similar to that we documented for Rod1/Art4 because they all contain consensus Ypk1 phospho-acceptor motifs (number indicated in parentheses) that are highly conserved among the sensu stricto species or have been detected as phosphorylated in phospho-proteomic studies, or both.

The second mechanism by which Ypk1-mediated phosphorylation impedes endocytosis is via inhibition of Fpk1 and Fpk2. Based on the stringent phospho-acceptor site preference we defined for Fpk1 [52], we examined its potential substrates and validated in vitro and in vivo that, aside from the flippases, a bona fide target of Fpk1 is yet another protein kinase, Akl1 [26]. Although considerably longer at its C-terminal end, Akl1 (1108 residues) is closely related in its kinase domain to protein kinases Ark1 (638 residues) and Prk1 (810 residues), which had been shown to modulate the dynamics of actin patch-mediated endocytosis [182]. We found that Akl1 phosphorylates and blocks the function of several actin patch-associated proteins [26]. We demonstrated that Fpk1 phosphorylates Akl1 at two conserved C-terminal sites, which both Ark1 and Prk1 lack, and that these modifications inhibit Akl1 [26]. Thus, under normal growth conditions, Fpk1-mediated inhibition of Akl1 prevents it from interfering with endocytosis. However, under conditions that upregulate TORC2-Ypk1 signaling, Fpk1 is inhibited by Ypk1-dependent phosphorylation, alleviating the Fpk1-mediated inhibition of Akl1, allowing Akl1 to phosphorylate and block the action of proteins needed for endocytic patch function [26]. 

So, overall, under stressful conditions that threaten PM integrity and activate TORC2-Ypk1 signaling (including sphingolipid limitation, heat shock, hypotonic conditions), the cell will not further compromise PM function by endocytic removal of PM proteins because Ypk1 action blocks α-arrestin function and prevents Fpk1 from inhibiting Akl1. Conversely, upon hypertonic shock, where TORC2-Ypk1 signaling is shut off rapidly, PM proteins can be removed as part of the clearance of the “excess” membrane created by cell shrinkage because α-arrestins remain fully functional and Fpk1 is able to prevent Akl1 from acting. Thus, by these mechanisms, TORC2-Ypk1 signaling adjusts the rate of endocytosis to meet the needs of the cell. 

### 5.5. Other Potential Targets

The MADS-box transcription factor Smp1, paralog of Rlm1 [183,184], was identified initially as a potential Ypk1 target because it was isolated as a dosage suppressor of the temperature-sensitive phenotype of *ypk1^ts^ ypk2Δ* cells [46]. It was identified independently as a likely Ypk1 substrate in our global screen [23] and confirmed to be an authentic substrate of Ypk1 in vivo [185]. Smp1 has been implicated both in response to hyperosmotic stress [184,186] and in sensitivity to the toxic effects of high exogenous Fe^3+^ [187] and, both Smp1 and Ypk1 are required for iron toxicity [187]. Preliminary microarray analysis [188] supports the conclusion that TORC2-Ypk1 signaling may be mechanistically coupled to iron metabolism by modulation of genes under Smp1 transcriptional control. Ypk1 may also contribute to translational control, given that phosphorylation of Ser232 in ribosomal protein S6 (Rps6) has been attributed, in part, to Ypk1 action [189]. In this regard, it has also been reported [190] that loss of Ypk1 function impedes translation, apparently due to depletion of eIF4G, an initiation factor essential for cap-dependent mRNA translation [191].

There is also circumstantial evidence that the actions of Ypk1 and/or Ypk2 have roles in various other aspects of yeast cell physiology, including dampening the damaging effects of reactive oxygen species (ROS) from both vacuolar and mitochondrial sources [192,193], as well as promoting autophagy in a Ca^2+^-dependent manner when cells are starved specifically for amino acids [194,195]. The mechanism by which Ypk1 regulates ROS is unclear, but somehow involves Ypk1 control of flippases and sphingolipids [192,193]. In addition, Ypk1 reportedly modulates the activity specifically of the Tpk3 isoform of PKA, by somehow upregulating expression of *PDE2*, a gene encoding the high-affinity 3’,5’-cyclic-AMP phosphodiesterase [193]. However, in none of these studies has the actual substrate phosphorylated by Ypk1 and/or Ypk2 that is responsible for the reported phenotypic observations been pinpointed.

Also, in a study of ligand-induced endocytosis of Ste2 [196], it was reported that “purified” Ypk2 (but not putative kinase-dead mutants) was able to phosphorylate in vitro a site (Ser357) in a fragment of the ATPase domain of the paralogous type I myosins Myo3 and Myo5, which serve as the motor proteins for actin-driven endocytosis, and that this modification promotes Ste2 internalization [197]. This conclusion seems suspect, however, given that it was not shown that modification of this site in vivo is Ypk2-dependent under any condition and given the more recent evidence, summarized in a preceding section, that Ypk1 and Ypk2 negatively regulate endocytosis. Moreover, this same site was well-documented long ago to be the target for the yeast Cdc42-dependent protein kinases Cla4 and Ste20 (homologs of mammalian p21-activated protein kinases / PAKs) [198]. Indeed, Ser357 in Myo3 and Myo5 lies in a sequence context that was shown by others to be the optimal consensus phospho-acceptor motif for Cla4 and Ste20 [136,199,200], and certainly not for Ypk1 and Ypk2 [23,136]. 

Another means to glean what aspects of cell function are under TORC2-Ypk1 control was a so-called chemical genetic approach [201]. Specifically, Tor2 was mutagenized and a derivative was isolated that is preferentially susceptible to inhibition by a chemical TOR inhibitor, the imidazoquinoline derivative NVP-BEZ235 [202]. This tool—the ability to selectively inhibit TORC2 action—was combined with a collection of deletion mutants to identify what processes, when eliminated, are especially deleterious to cell growth and survival when TORC2 action (and presumably Ypk1 activity) is limiting. This analysis suggested some connection between TORC2 action and the pentose-phosphate pathway [202], in keeping with the growth-promoting role of TORC2 and the demand for NADPH in many cellular anabolic reactions. Indeed, two of the core reactions of yeast sphingolipid biosynthesis require NADPH as a cofactor: conversion of 3-ketodihydrosphingosine to dihydrosphingosine (catalyzed by Tsc10) and conversion of dihydrosphingosine to phytosphingosine (catalyzed by Sur2). However, in this study, no Ypk1 substrate was identified.

Similarly, use of TOR inhibitors implicated TORC2-Ypk1 signaling in regulation of actin filament formation that is somehow required for yeast cell survival in response to low levels of DNA damage [203]. Again, however, no Ypk1 substrate was identified. In contrast, in another study in which a yeast strain was engineered in which TORC2 could be specifically inhibited by another imidazoquinoline-derived TOR inhibitor (NVP-BHS345), it was found using phosphoproteomics that action of the Ypk1 substrates Fpk1 and Fpk2 was required to see the inhibition of endocytosis after acute inhibition of TORC2 [34], in agreement with findings already presented above [26]. 

Using an alternative way to block TORC2 activity, namely shift of the temperature-sensitive *tor2-21^ts^* allele, it was found [132] using microarray analysis that approximately half of the genes upregulated following TORC2 inhibition are under the control of the calcineurin-activated transcription factor Crz1 [204]. Likewise, using another chemical genetic tactic, namely when a strain bearing an analog-sensitive allele [205] of Ypk1 (Ypk1-as) was treated with inhibitor, microarray analysis also revealed an increase in Crz1-dependent gene transcription [29], suggesting that TORC2-Ypk1 action somehow leads to downregulation of calcineurin activity. There is evidence that the Ca^2+^ needed to activate calcineurin is admitted to the cytosol via the ER- and PM-localized calcium channel Mid1 [206], suggesting that Mid1 function is blocked by Ypk1-dependent regulation. However, this regulation must be indirect because Mid1 itself lacks Ypk1 consensus phospho-acceptor motifs.

Of course, given the evidence that TORC2 may regulate Pkc1 under certain stress conditions [207], potential Pkc1 targets might also emerge from studies in which TORC2 function is inhibited. Indeed, in the *avo3∆C TOR1-1* strain, inhibition by rapamycin causes a G2-M cell cycle arrest [15] similar to what is observed in Pkc1-deficient cells [38]. Pkc1 and its substrates are discussed further below.

## 6. Structure, Function and Regulation of Pkc1

Like *YPK2*, the *PKC1* gene was first identified and isolated via its ability to hybridize to cDNA probes encoding isozymes of rat PKCs [38]. However, the *PKC1* locus was also identified genetically in several ways: (a) as the *stt1* mutation [208], one of 14 distinct complementation groups that conferred both temperature-sensitive growth and elevated sensitivity to the killing action of staurosporine, a bis-indole anti-fungal agent that acts as an ATP mimic and thus is able to block the active site of protein kinases [209]; (b) as several allelic temperature-sensitive mutations (*cly5, cly7, cly15*) that caused spontaneous cell lysis when shifted to the restrictive temperature [210,211]; and, (c) as the *hpo2* mutation, which displayed enhanced cell killing by the cell swelling caused by shifting cells to hypotonic medium [212]. Moreover, although a *pkc1∆* mutation is lethal [38], and cells depleted of Pkc1 arrest cell division with small buds and at a point subsequent to DNA replication [38,210], *pkc1∆* cells are able to grow on medium containing an osmotic support (1 M sorbitol), but with grossly abnormal morphology and rapidly lyse if then shifted to normal medium [210,213]. In marked contrast, although the temperature-sensitive growth defect of *ypk1^ts^ ypk2∆* cells is ameliorated on sorbitol plates [46], the inviability of *ypk1∆ ypk2∆* cells is not [214]. Examination of Pkc1-deficient cells by electron microscopy (EM) revealed that they have thinner cell walls (with less β-glucan and phosphomannoprotein) and develop large holes in the wall mainly at the bud tip [215], the primary site of polarized growth [216]. Collectively, these observations strongly indicated that loss of Pkc1 function causes a major defect in cell wall integrity and suggested that an important function of Pkc1 is to control the process of cell wall remodeling during growth, which is indeed the case, as described further below. 

### 6.1. Activation Loop Phosphorylation by Pkh1 and Pkh2 

The first suggestive evidence that Pkc1 was a downstream target of Pkh1 and Pkh2 was the observation that the phenotypes of a *pkh1(D398G)^ts^ pkh2* strain resembled mutants defective in Pkc1 function (or in the function of downstream components of the cell wall integrity signaling pathway that it initiates) [69]. Initial biochemical studies showed that Pkh2 was able to phosphorylate Pkc1 in vitro, that the site of phosphorylation (Thr983) (Figure 2) lies in its activation loop within a highly conserved motif very similar to that phosphorylated by mammalian PDK1 in its targets (Figure 4), that Pkc1 activity was markedly reduced in *pkh1(D398G)^ts^ pkh2* cells shifted to the restrictive temperature, and that phosphorylation of Pkc1 at Thr983 is essential for its function [69]. These observations were amply corroborated subsequently [47,99]. Furthermore, as mentioned earlier, hyperactive alleles of Ypk1 and Ypk2, namely Ypk1(D242A) or Ypk2(D239A), are able to rescue the inviability of cells deficient in TORC2 function [22,67,68]. However, such alleles do not rescue the temperature-sensitive lethality of *pkh1(D398G)^ts^ pkh2* cells [67], consistent with the fact that Pkc1 is also an essential protein that requires activation by Pkh1- and Pkh2-mediated phosphorylation. 

### 6.2. C-Terminal Phosphorylation by TORC2

It was recognized quite some time ago [47] that the short C-terminal extension in Pkc1 contains sequences very similar to the conserved turn and hydrophobic motifs found in other yeast and mammalian AGC kinases that are now known to be phosphorylated by TORC2. Shortly thereafter, genetic data linked TORC2 function to the regulation of Pkc1 and its control of cell wall integrity. Mutants carrying temperature-sensitive alleles in different regions of the essential TORC2 component Avo3/Tsc11 displayed cell wall defects, as evidenced by characteristic rescue of their cell lysis defect by 1 M sorbitol, their decreased trypan blue staining (diagnostic of less phosphomannoprotein), and their increased sensitivity to killing by digestion by cell wall-degrading enzymes; moreover, *PKC1* was isolated as a dosage suppressor of some of these *avo3^ts^* alleles [217]. Yet, according to cumulative data compiled at the SGD, and unlike the hydrophobic motif (Thr662) in Ypk1, phosphorylation at the hydrophobic motif (Ser1143) in Pkc1 has not been detected in any global phosphoproteomic study. However, it has been reported that, in response to a non-physiological concentration (10 mM) of exogenous methylglyoxal, TORC2-dependent phosphorylation at both the turn motif (Thr1125) and Ser1143 is markedly stimulated [207]. Moreover, lack of phosphorylation at the turn motif site reduced phosphorylation at the hydrophobic motif site, and vice-versa [207], reminiscent of the interdependencies we have found among the C-terminal TORC2 phosphorylation sites in Ypk1 [39]. On the other hand, the biological significance of this response to such a high extracellular concentration of this very reactive and toxic aldehyde is questionable. Moreover, unlike a *pkc1Δ* mutant, which is unable to grow in the absence of an osmotic stabilizer (e.g., 1 M sorbitol), cells expressing a Pkc1(T1125A S1143A) mutant as the sole source of this protein kinase, grew in normal medium without sorbitol [207]. Thus, in Pkc1, phosphorylation of both its turn and hydrophobic motifs is dispensable for the growth of yeast cells on normal medium, whereas that is not the case for Ypk1 or Ypk2. In *ypk1∆ ypk2∆* cells, expression of either Ypk1(S664A) or Ypk2(S641A), which cannot be phosphorylated at their turn motif, support only very poor growth compared to the same cells expressing either WT Ypk1 or WT Ypk2 [39,67]. So, it remains to be determined whether any physiologically relevant stress(es) or signal(s) modulate Pkc1 function by affecting its TORC2-mediated phosphorylation.

### 6.3. Interaction with Rho1-GTP 

The *S. cerevisiae* genome encodes five members of the Rho sub-family of Ras-related small GTPases (and Cdc42 is also closely related). Single deletions of each of the other four *RHO* genes are tolerated, whereas a *rho1∆* mutation is lethal [218,219]. Based on the phenotypes of a variety of conditional alleles, Rho1 has roles in the establishment of cell polarity, in organization of the actin cytoskeleton, and in bud morphogenesis. Rho1 (209 residues) contains a C-terminal CaaX box, which is subject to geranylgeranylation on the S of the Cys residue, followed by proteolytic cleavage on the C-side of the prenylated Cys and methylation of the now-exposed carboxyl group of the prenylated Cys, providing a lipophilic anchor to the PM [220]. Moreover, aside from a single Glu residue, the C-terminal portion of Rho1 is composed exclusively of residues with neutral and positively-charged side chains (-RASLMGKSKTNGKAKKNTTEKKKKKCVLL), facilitating electrostatic interaction with the head groups of negatively-charged phospholipids [221]. At the PM, Rho1 is prominently localized at sites of growth, such as the incipient budding site, the bud tip, and the bud neck during cytokinesis [222,223]. The guanine nucleotide exchange factors (GEFs) responsible for production of the GTP-bound state of Rho1 are Rom1 and its paralog Rom2 and a less-related Rho1 GEF Tus1. All three of these GEFs have been linked to activation of Pkc1 and the cell wall integrity pathway under its control [214,224,225]. Rho1-GTP is downregulated by the GTP-activating proteins (GAPs) Sac7 and its paralog Bag7, Lrg1 and Bem2, as well as by the guanine nucleotide dissociation inhibitor Rdi1, which extracts prenylated Rho1 from the membrane. In addition to direct interaction with and activation of Pkc1, the β(1→ 3)-glucan synthase Fks1 and its paralog Gsc2/Fks2 both bind and are activated by Rho1-GTP [226,227]. The interplay between the Rho1 GEFs and GAPs, whose activities are, in turn, dictated by their state of phosphorylation [228], determines when and where Rho1-GTP will be available to activate its effectors. Thus, active Rho1 can promote distinct signaling outputs under different conditions. 

In any event, crystal structures and other biochemical evidence document that Rho1-GTP associates exclusively and highly specifically with the α-helical Hr1 domains in all PKN members [229,230], including yeast Pkc1 [231]. Indeed, it was found that Pkc1 co-immunoprecipitates with Rho1 from yeast cell extracts, that recombinant Rho1 associates with Pkc1 in vitro in a GTP-dependent manner, and that the binding of Rho1-GTP is a prerequisite for Pkc1 activity to respond to stimulation by PtdSer [57], as discussed further in the next section. These findings suggest that interaction of the tandem N-terminal Hr1 domains in Pkc1 with Rho1-GTP (Figure 2), aside from tethering Pkc1 to the PM, also enhances its ability to be responsive to its lipid modulators, presumably by causing conformational changes that expose the necessary binding sites and/or by bringing the enzyme to the membrane where they reside.

### 6.4. Modulation by PtdSer and DAG 

**Figure 7:**
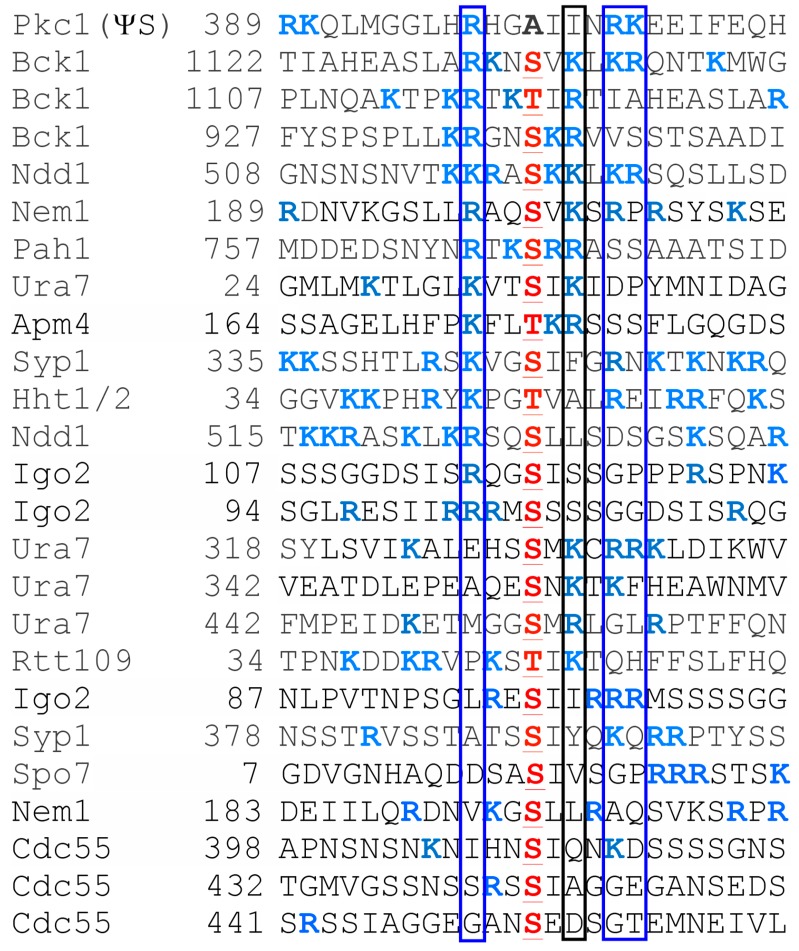
Reported sites of Pkc1 phosphorylation. The evidence for Pkc1 phosphorylation of the indicated site (red) in the gene products listed (position of first residue in the sequence shown indicated on the left) can be found described in the following citations: Amp4 [237]; Bck1 [215,238]; Cdc55 [239]; Hht1 and Hht2 [240]; Igo2 [239]; Ndd1 [241]; Nem1 [233]; Pah1 [233]; Rtt109 [240]; Syp1 [242]; and, Ura7 [233,243].

Shortly after the *PKC1* gene was cloned, a C-terminally epitope-tagged version was expressed in yeast and recovered from cell extracts by immuno-precipitation under non-denaturing conditions, presumably with Rho1 still bound, although that was not examined at the time [232]. Using these preparations for enzyme assays, calf thymus histones and myelin basic protein as surrogate substrates (because no physiologically relevant substrate for Pkc1 was then known), and Mg^2+^ and [γ-^32^P]ATP, it was found that addition of Ca^2+^, PtdSer, and DAG synergistically stimulated Pkc1 autophosphorylation as well as incorporation of phosphate into these substrates [232]. Moreover, addition of a synthetic peptide that mimics the pseudosubstrate sequence in Pkc1 (except that the Ala was changed to a Ser) (Figure 7), namely GGLHRHG(A→ S)IINRK, markedly reduced both autophosphorylation and substrate phosphorylation [232]. Quite recently, this issue was revisited for protein substrates thought to be bona fide in vivo targets of Pkc1. For peptides containing sites from Pah1 (PtdOH phosphatase), Nem1 (catalytic subunit of a protein phosphatase that dephosphorylates Pah1), and Spo7 (regulatory subunit of the protein phosphatase that dephosphorylates Pah1), the efficiency of their phosphorylation was not enhanced by the presence of lipids [233]. However, when the same full-length proteins (Pah1, Nem1, and Spo7) were used as substrates, PtdSer and DAG were required for their phosphorylation, with PtdSer having a greater effect than DAG [233]. Moreover, in an in vitro liposome binding assay, the presence of PtdSer enhanced Pkc1 retention in a dose-dependent manner and, in *cho1∆/pss1∆* cells (which lack PtdSer synthase), the degradation of Pah1, which is thought to be a Pkc1-dependent process, was attenuated, consistent with a role for PtdSer in regulating Pkc1 function in vivo [233]. 

In this same regard, Pkc1 contains an apparent C2 domain (Figure 2), which is net acidic (20 D and E, 16 R and K) and with three of the five residues equivalent to those that chelate Ca^2+^ in synaptotagmin conserved (E234, D277, and D280) and the other two positions neutral (M228 and V279) [234]. C2 domains with these features have been amply demonstrated to mediate binding to anionic lipids on the cytosolic surface of the PM, especially PtdSer and phosphoinositides (PIPs) in a Ca^2+^-dependent manner [235,236]. Thus, as in other C2 domain-containing proteins, it is interaction with this region of Pkc1 that presumably mediates the observed stimulatory effect of PtdSer. Furthermore, unlike the inviability of *pkc1∆* cells, the temperature-sensitive lethality of each of three different *pkc1^ts^* alleles [Pkc1(N834I), Pkc1(L887S), and Pkc1(P1023L)] could be suppressed on medium containing 25 mM CaCl_2_ [213], at least consistent with the stimulatory effect of the presence of Ca^2+^ on Pkc1 activity in vitro that we observed [232].

Pkc1 also possesses apparent tandem Cys-rich C1 domains (Figure 2). In other proteins that contain such tandem, Cys-rich Zn^2+^-binding C1 domains, the only natural lipid with which they associate is DAG [244,245]. Hence, as in other C1 domain-containing proteins, it is interaction with this region of Pkc1 that presumably mediates the observed stimulatory effect of DAG. A recent claim [246] that it is the C1 domains of Pkc1 that mediate its association with both Rho1-GTP and PtdSer, and that DAG has no effect, seem strikingly at odds with the clear-cut precedents and prior observations of others cited above. Indeed, it is very well established that in both PKCs and PKNs, once the catalytic domain has been properly folded via its activation loop phosphorylation, it is the binding of the C2 domain to acidic phospholipids (usually in Ca^2+^-dependent manner) and binding of the C1 domains to DAG that act in a concerted manner to activate these protein kinases at the PM [247,248]; membrane association stabilized by the synergistic actions of these three factors (Ca^2+^, PtdSer, and DAG), a form of coincidence detection, induces the conformational changes necessary to release the pseudosubstrate sequence from the catalytic pocket, allowing the enzyme to phosphorylate targets [247,248]. Thus, in the case of Pkc1, for it to act at the PM, it seems likely that sufficient Rho1-GTP must be generated to recruit it and sufficient concentrations of Ca^2+^, PtdSer, and DAG must be present there to activate its catalytic function.

## 7. Substrates of Pkc1

In the past, because of its original designation, it was assumed that the substrate specificity of yeast Pkc1 would recapitulate that of its apparent higher cell counterparts and, for that reason, often commercially-available preparations of mammalian PKCs were used to define “Pkc1” sites in other yeast proteins, instead of bona fide purified and Rho1-GTP-activated Pkc1. Moreover, for many of these putative sites, the phosphorylated residue was not unequivocally identified nor was its modification demonstrated to be Pkc1-mediated in vivo. So, many of these assignments need to be verified. Furthermore, spatial restriction of Pkc1 and/or of its required cofactors will dictate in which subcellular compartment Pkc1 can act and therefore what substrates it can phosphorylate. In this regard, it has been reported that, like Rho1 itself, the bulk of full-length Pkc1 localizes to sites of polarized growth [249], consistent with its known function in maintenance of cell wall integrity signaling. Moreover, using a fragment-based approach, it was found that a segment containing the tandem Hr1 domains localized to the bud tip, whereas a portion containing the tandem C1 domains localized to the PM all around the cell periphery [249]. However, certain fragments displayed prominent nuclear localization, which lead to the identification of two functional nuclear localization signal (NLS) elements [249]—one SV40 T-antigen-like (554-KKKRTVP-560) and one bipartite nucleoplasmin-like (808-KHKKRAAKRRKVSL-821) [250]. Yet, full-length Pkc1 does not localize to the nucleus, raising the possibility that Pkc1 undergoes nucleocytoplasmic shuttling in which its rate of nuclear export keeps pace with its rate of import; and, that hypothesis led to the identification in Pkc1 of a functional nuclear export signal (NES; 51-**L**EY**L**EDS**L**KK**L**R**L**-63) [249] of the Leu-rich Crm1/Xpo1-dependent class [251]. This shuttling may explain why certain reported Pkc1 substrates (Figure 7) are located in the nucleus (see further below); however, since the nucleus appears to lack Rho1 and its content of Ca^2+^, PtdSer, and DAG is uncertain, it raises the question of what factors, if any, allow Pkc1 to be active in this compartment. It is also of some potential concern that the conclusions of this fragment-based approach were not corroborated by installing in full-length Pkc1 substitution mutations that ablate the folding and/or the ligand-binding residues in each of the corresponding motifs (Hr1 domains, C2 domain, etc.) and examining the consequences. To further confound matters about the importance of localization and the role of modulatory factors, it has been reported that a cloned fragment that encodes only the C-terminal catalytic domain of Pkc1, which was isolated on the basis of its complementing the temperature-sensitive lysis defect of a *pkc1^ts^* allele (*cly15*), is also able, at least when overexpressed from a *GAL* promoter on a plasmid, to rescue the growth and, quite surprisingly, the morphology defects of a *pkc1∆* null mutant [210]. For these reasons, much is still uncertain about how, why, and where in the cell Pkc1 is active and carries out its multifarious functions. 

### 7.1. Cell Wall Integrity Pathway

The lethal cell lysis defect of Pkc1-deficient cells provided a strong positive selection for both genomic extragenic suppressors and plasmid-borne dosage suppressors that restored growth and viability as a means to identify genes that function downstream of, but within the same pathway, as Pkc1. In this way, dominant alleles mapping to the *BCK1* (Bypass of C Kinase) gene were identified [215]. In the immediately preceding years, the concept of multi-tiered protein kinase cascades involved in cellular responses to various stresses and extracellular stimuli had emerged from studies in both yeast and mammalian cells [252,253,254,255]. The sequence similarity of Bck1 to the MAP kinase kinase kinase (MAPKKK) of the mating pheromone response pathway (Ste11) and to a MAPKKK in the HOG pathway (Ssk2), immediately suggested that one function served by Pkc1 was as the upstream activator (i.e., a MAPKKKK) of such a cascade, in the same way that Ste20 serves as the MAPKKKK in the mating pheromone response pathway [256,257] and in the Sho1 branch of the HOG pathway [258,259]. Indeed, a Pkc1-initiated pathway that culminates in activation of the MAPK Slt2/Mpk1 was methodically mapped out by genetic and biochemical methods, as reviewed in detail elsewhere [260,261]. Consistent with its role as an activator of Bck1, it has been reported that Pkc1 selectively phosphorylates Bck1 in vitro at three sites (Figure 7) and, that in the absence of Bck1, Slt2/Mpk1 is not active [215,238]. One of the reported sites in Bck1 matches well with features of the pseudosubstrate sequence in Pkc1: L at −5; R at −3; I/V at +1; and, R or K at both +4 and +5 (Figure 7). Indeed, as we observed [232], and in the hands of at least two other groups, peptides in which the Ala within the pseudosubstrate sequence is mutated to either Ser or Thr serve as efficient in vitro substrates for Pkc1 purified from yeast [262,263]. Moreover, mutational alteration of these peptides revealed that replacing the Ile at position +2 (with respect to the phosphorylated residue) with Arg enhanced phosphorylation of the peptide and its binding to Pkc1 [262,263]. Conversely, substitution of Ala for the Arg at −3 and for the basic residues at +4 and +5 eliminated phosphorylation of the peptide, and substitution of Arg −3 alone had a stronger effect (100-fold poorer binding) than substitution of both basic residues at +4 and +5 alone (10-fold reduction in binding) [262]. On this basis, it appears that the minimal phospho-acceptor site preference for Pkc1 is R (or K) at −3 and R (or K) at +2 [263]. The other two reported sites in Bck1 fit this consensus (Figure 7).

Unlike *pkc1∆* mutants, *slt2∆/mpk1∆* cells are viable under normal growth conditions [264], indicating that Pkc1 action is required for other cellular processes in addition to its function in stimulating the cell wall integrity (or, perhaps, better called the “cell wall damage repair”) pathway [228]. As one means to detect additional potential Pkc1 substrates, MS analysis was conducted [265] comparing normal cells to those overexpressing a constitutively-active Pkc1 allele, Pkc1(R398A R405A K406A), dubbed Pkc1*, that is hyper-active because critical residues necessary for the inhibitory function of its pseudosubstrate sequence have been mutated [263]. Numerous phosphopeptides representing both validated and potentially new Slt2/Mpk1 targets were identified by this procedure; but, surprisingly, no phosphopeptides corresponding to Bck1 or to any other of the putative Pkc1 substrates reported by others (Figure 7) were recovered by this in vivo approach [265]. 

Because Slt2/Mpk1 function, which acts, in part, by upregulating the expression of genes for cell wall synthesis and remodeling enzymes [260,261], is required only under conditions that subject the cell wall to damage or stress (treatment with cell wall-degrading enzymes; exposure to cell wall-perturbing dyes, including Calcofluor White and Congo Red; hypotonic conditions), there must be a mechanism(s) to ensure the transcription of the same sets of genes that do not require Slt2/Mpk1 action. One such back-up pathway appears to be dependent on the function of calcineurin, the Ca^2+^-calmodulin-dependent protein phosphatase, because loss of calcineurin function is tolerated in otherwise WT cells, but is lethal in *pkc1^ts^* and *slt2∆/mpk1∆* mutants [266], suggesting that calcineurin and Pkc1-Slt2/Mpk1 perform independent, but physiologically related, functions. In any case, it has been shown that at least the gene for one β(1→3)-glucan synthase, Gsc2/Fks2, is under their joint control [267].

### 7.2. Nuclear Targets 

Given the evidence discussed above that Pkc1 undergoes nucleocytoplasmic shuttling [249], it is perhaps not unexpected that substrates for Pkc1 that are confined to the nucleus have been described. Moreover, given the original observation that Pkc1-deficient cells display a cell cycle-specific arrest at the G2-M transition [38], it is perhaps satisfying that one nuclear target reportedly phosphorylated by Pkc1 is the Ndd1 subunit of the Mcm1-Fkh2-Ndd1 transactivator complex [241], which is part of the machinery that controls the periodic expression of B type cyclins (Clbs) in yeast. One of two reported sites in Ndd1 matches well the features of the minimal Pkc1 phospho-acceptor site consenus defined above (Figure 7). Absence of phosphorylation of Ndd1 at these sites causes late cell cycle defects due to changes in the timing of the expression of the *CLB2-CLB5* cluster on chromosome XVI [241].

In addition to transcription, evidence has been obtained that Pkc1 phosphorylates two nuclear targets that are necessary for efficient re-assembly of chromatin following DNA replication [240]. For this process, histone H3 (Hht1 and Hht2 in yeast) is phosphorylated at Thr46, which promotes its acetylation at Lys57. Under conditions of replicative stress, Pkc1 appears responsible for H3 T46 phosphorylation and Pkc1, by reportedly phosphorylating Thr46 (by coincidence at the same residue number) in the histone acetyltransferase Rtt109, also stimulates acetylation of H3 K57 [240]. The site in Hht1 (Hht2 is identical in sequence) matches some features of the pseudosubstrate sequence in Pkc1, whereas it matches the putative site in Rtt109 much less so (Figure 7). It would be unprecedented for an AGC kinase family member to have such a broad or lax phospho-acceptor sequence preference; hence, the conclusion that Thr46 in Rtt109 is a direct target of Pkc1 needs further corroboration.

### 7.3. Modulation of Triacylglycerol Synthesis

As described above, for synthesis of glycerophospholipids, PtdOH generated by fatty acylation of Glo3P can react with CTP to form CDP-DAG and pyrophosphate; the CDP-DAG reacts with inositol, glycerol or serine, releasing CMP and forming PtdIns, PtdGlo and PtdSer. PtdSer can be converted (by decarboxylation) to PtdEth, which can, in turn, be N-trimethylated to form PtdCho. However, PtdEth and PtdCho can also be generated by an alternate route (Kennedy pathway). Ethanolamine and choline are converted to P-Eth and P-Cho by cognate ATP-dependent kinases; P-Eth and P-Cho then react with CTP to form CDP-Eth and CDP-Cho, which can react with DAG, releasing CMP and forming PtdEth and PtdCho. Thus, during active cell growth, both routes of glycerophospholipid production place a high demand on the supply of CTP and, not surprisingly therefore, the major yeast CTP synthetase isozyme (Ura7), which forms CTP by catalyzing the ATP-dependent transfer of the amide nitrogen of Gln to UTP, is subject to metabolic regulation [268].

Moreover, in addition to its biosynthetic role in the Kennedy pathway, DAG also has a different physiological function: when fatty acyl-CoAs are in excess of the needs of the cell, they can be used to esterify DAG, forming triacylglycerol for storage in lipid droplets. Because DAG has these two distinct metabolic fates, it is also not surprising that Pah1, the specific phosphatase that generates DAG from PtdOH [269], would also be subject to tight regulation to maintain lipid homeostasis because Pah1 action directly controls the relative cellular concentrations of its substrate PtdOH and its product, DAG. The relative levels of these intermediates will, in turn, affect the rates of membrane biogenesis versus lipid droplet formation.

Indeed, the subcellular location/membrane binding [270], rate of degradation [271], and PtdOH phosphatase activity of Pah1 [272] are regulated by phosphorylation and dephosphorylation. In this regard, it has been reported, over time, that Pah1 is a target for phosphorylation by Cdk1 (Cdc28-Clb), by PKA (although, which yeast isoform, Tpk1, Tpk2, and Tpk3, was not addressed), by Cdk5 (Pho85-Pho80), by casein kinase II (Cka1-Cka2-Ckb1-Ckb2 heterotetramer in yeast) [273], and also by Pkc1 [233]. Dephosphorylation of Pah1 stimulates its catalytic activity and facilitates its association with membranes, wherein its substrate resides; dephosphorylation of Pah1 is mediated by an ER-associated heterodimeric phosphoprotein phosphatase Nem1-Spo7. Like Pah1, both Nem1 and Spo7 are purported substrates for many of the same protein kinases, including Pkc1 [233], although there is also compelling evidence that activating phosphorylation of Nem1-Spo7 is controlled by a protein kinase that is negatively regulated by TORC1 [274]. Likewise, it has been reported that the activity of Ura7 CTP synthetase is also stimulated by Pkc1-mediated phosphorylation [165,233]. Indeed, a site in Pah1, a site in Nem1, and a site in Ura7 all match well the elements of the minimal consensus defined by mutagenesis of the pseudosubstrate sequence and in comparison to the mapped sites in Bck1 (Figure 7). By contrast, three other reported sites in Ura7 and other reported sites in Spo7 and Nem1 do not. Assuming the Pkc1-mediated phosphorylation of Pah1, Nem1, and Ura7 has some collective physiological consequence in the cell, then, with respect to overall control of lipid homeostasis, Ypk1 and Pkc1 would share a related biological function, albeit at very different entry points of the glycerophospholipid metabolic network.

### 7.4. Other Potential Targets

Nem1-Spo7 is not the only phosphoprotein phosphatase reportedly regulated by Pkc1-mediated phosphorylation. The Cdc55-bound form of phosphoprotein phosphatase 2A (PP2A) and its small protein regulators, the so-called “endosulfins” (Igo1 and Igo2 in yeast), have important roles in controlling the timing of mitotic entry and exit [275]. The protein kinase Rim15, the yeast ortholog of Drosophila Greatwall and human GWL/MASTL [276], is responsible for installing the phosphorylations of Igo1 and Igo2 that make them inhibitors of Cdc55-PP2A [277], thereby sustaining Cdk1-mediated phosphorylations and promoting mitotic entry. How that inhibition is alleviated to promote mitotic exit has not been completely clear, although in other systems, the Cdc55-PP2A equivalent itself is the enzyme responsible for dephosphorylating the phosphorylated endosulfines during M phase exit [278]. In this context, it was observed that, in yeast cells expressing hyperactive Pkc1*, they could not hold cell cycle arrest at a mitotic checkpoint. Therefore, Pkc1-dependent modifications in such cells were examined by MS and changes were observed in Cdc55 and Igo2, which seemed to promote dissociation of Igo2, and the putative Pkc1 sites were mapped. However, mutation of the apparent Pkc1-dependent phosphorylation sites on Cdc55 and Igo2, none of which match well with what appears to be the minimal consensus Pkc1 phospho-acceptor motif (Figure 7), did not cause any detectable defects in mitotic progression [239]. 

Another reported Pkc1 substrate is the N-terminal F-BAR domain-containing and C-terminal Muniscin/Mu homology domain (MHD)-containing protein Syp1, which was initially reported to localize prominently at the bud neck and was implicated in the processes required for disassembly of the septin ring [279]. Syp1 is a highly phosphorylated protein [280,281,282]; eight of the phospho-sites detected in Syp1 fit the minimal -T/S-P- consensus for the major cell cycle driver Cdk1/Cdc28 [136,283] and five fit the minimal consensus sequence (-R-x-x-S/T-) determined for the bud neck-localized protein kinase Gin4 [136,284]. However, others find that Syp1 localizes predominantly to cortical puncta and functions as an endocytic adaptor that is involved in cargo selection and negative regulation of Las17 (yeast WASp)-Arp2/3 complex activity [285,286] during the early stages of actin patch formation for clathrin-dependent endocytosis [287]. Although eukaryotic proteins with such apparently disparate functions certainly exist, it is nonetheless a bit hard to reconcile these two rather distinct roles attributed to Syp1 mechanistically. Also, contrary to the findings implicating Syp1 in septin ring disassembly and in endocytic actin patch function, is a study claiming that Syp1 is phosphorylated in a Rho1- and Pkc1-dependent manner to promote septin collar assembly [242]. The context around only one of the two sites reportedly phosphorylated by Pkc1 in Syp1 resembles well the pseudosubstrate sequence in Pkc1 and other likely Pkc1 sites, whereas a putative Pkc1 site in the cargo-binding clathrin-associated adaptor Apm4 is a better match [237] (Figure 7).

There is also a report that Pkc1 action is required for processing body (P-body) assembly and for mRNA decay at the step of deadenylation of the 3’-polyA tail under nutrient poor conditions [288]. Unfortunately, in this study, no Pkc1 substrates involved in either of these processes were identified. Finally, despite all of the observations that *PKC1*-regulated processes affect the actin cytoskeleton [67,193,214,289], no direct Pkc1 target intimately associated with the control of actin filament assembly and dynamics per se has yet been reported.

## 8. Cross-Talk between Ypk1- and Pkc1-Dependent Signaling

One early indication that TORC2 action involves the integration of different cellular signaling pathways was the isolation of temperature-sensitive *tor2* alleles that fell into distinct phenotypic classes and the isolation of corresponding multicopy suppressors that could rescue one class, but not another [290]. Among the suppressors of a so-called Class A *tor2* mutant were genes encoding components of the Pkc1 pathway, including the Rho1 GEF Rom2 and Pkc1 itself, whereas genes encoding enzymes of phosphoinositide metabolism (e.g., the PtdIns4P 5-kinase Mss4) were among the suppressors of a so-called Class B *tor2* mutant [290]. These findings suggested that the functions of Tor2, and thus TORC2, involve at least two interrelated signaling pathways.

The first specific indication that Ypk1-mediated processes and Pkc1-mediated processes are intertwined also came from genetic observations. Among the dosage suppressors of *ypk1^ts^ ypk2∆* cells that we isolated was *EXG1* (the gene for a major exoglucanase) and among the extragenic suppressors created by transposon insertion were loss-of-function mutations in *KEX2* (the gene for an endoprotease in the *trans*-Golgi compartment necessary for the processing and proper trafficking of many proproteins and zymogens, including Exg1), suggesting that damage to the cell wall might bypass the need for full Ypk1 function by upregulating Pkc1 function and/or the cell wall integrity pathway [46]. Indeed, we found that the viability of *ypk1^ts^ ypk2∆* cells could also be maintained by *PKC1* overexpression or by an activated allele (*BCK1-20*) of the downstream protein kinase and, conversely, that a *slt2∆/mpk1∆* mutation was lethal in *ypk1^ts^ ypk2∆* cells [46], suggest that the processes Ypk1 controls to maintain PM homeostasis work in parallel with the Pkc1-controlled pathway for maintenance of cell wall integrity.

Likewise, it was shown independently by others that, like Pkc1-deficient cells, Ypk-deficient cells have actin defects and reduced ability to activate Slt2/Mpk1 and, conversely, that overexpression of the Rho1 GEF Tus1, of Rho1 itself, or upregulation of the *PKC1*-controlled MAPK pathway suppressed the growth and actin defects of Ypk-deficient cells [214]. Similarly, it has been found [34] that the TORC2-Ypk1-regulated protein kinase Fpk1 is needed to control polarization of endocytic actin patches, in agreement with other recent results [26], and, furthermore, that Ypk1 influences Pkc1 activity through effects on the PM localization of the Rho1 GEF Rom2, which depends on sphingolipid synthesis and Fpk1 [34], both of which are under TORC2-Ypk1 control, reinforcing the important links between TORC2-Ypk1 signaling and Pkc1, especially at the level of effects on Pkc1 mediated via Rho1.

At the mechanistic level, other recent evidence is also compatible with coupling between Ypk1 and Pkc1 signaling occurring at the level of the supply and/or localization of Rho1-GTP. First, among the suppressors of the temperature-sensitivity of both *lst8∆* and *tor2-21^ts^* cells were loss-of-function mutations in the gene for the Rho1 GAP Sac7, suggesting that increasing the steady-state supply of Rho1-GTP may, in turn, upregulate Pkc1 function, which is then responsible for the rescue [291]. Second, the PM lipid most responsible for PM recruitment of Rho1 under normal conditions is PtdIns4,5P2 [221]; however, it has been reported that, under conditions of membrane stress (hypotonic shock and myriocin treatment), PM PtdIns4,5P2 is acutely degraded, but that Rho1 is nevertheless retained at the PM because activation of TORC2-Ypk1 signaling somehow causes an alternative acidic glycerophospholipid—namely, PtdSer—to be accumulated in the inner leaflet of the PM [292]. It is further claimed [292] that the observed increase in PtdSer somehow arises from preventing Fpk1-mediated stimulation of the Lem3-dependent flippases Dnf1 and Dnf2 [52,284]. Third, it was recently shown that Pkc1 function is required for repolarization of the actin cytoskeleton after the application of a heat shock stress, a condition that also activates Ypk1 [30], that actin repolarization was greatly impaired in a *cho1∆* mutant (which lacks PtdSer synthase and thus can only produce this lipid via the Kennedy pathway), and that physical interaction of Rho1 with Pkc1 is also required for this recovery [293]. Thus, PtdSer-dependent recruitment of Rho1 and its subsequent ability to activate Pkc1 contribute to cell survival in response to various stresses. Moreover, since PtdSer itself is a positive effector of Pkc1 catalytic activity [232,233], it may further contribute to optimal Pkc1 function directly.

## 9. Prospectus

We anticipate that further investigation of the network of biochemical processes under the control of TORC2 and its effector protein kinases will continue to serve as an informative model for investigating molecular mechanisms in signal transduction and in the cell biology of stimulus-induced metabolic regulation.
